# Supplementary material for: Genome-wide association study of 23,500 individuals identifies 7 loci associated with brain ventricular volume
Source: Nat Commun. 2018 Sep 26;9:3945. doi: 10.1038/s41467-018-06234-w (PMC6158214; doi:10.1038/s41467-018-06234-w)
Supplement: Supplementary file 1 — Supplementary Information [file 41467_2018_6234_MOESM1_ESM.pdf]

Supplementary Information

Genome-wide association study of 23,500 individuals identifies 7 loci associated with brain ventricular volume

Vojinovic et al.

Content

Supplementary Note 1: Participating studies 4

Supplementary Note 2: Funding sources 10

Supplementary Figure 1. An overview of study design 15

Supplementary Figure 2A. Manhattan plot for stage 1 genome-wide association (GWA) meta-analysis 16

Supplementary Figure 2B. Quantile-quantile plot for stage 1 GWA meta-analysis 16

Supplementary Figure 3. Quantile-quantile plot for stage 3 GWA meta-analysis 17

Supplementary Figure 4. Plot of effect size and mean age of cohorts with homogeneous phenotyping method for lead variant at 10p12.31 locus 18

Supplementary Figure 5. Plot of effect size and mean age of cohorts with homogeneous phenotyping method for lead variant at 3q28 locus 19

Supplementary Figure 6. Plot of effect size and mean age of cohorts with homogeneous phenotyping method for lead variant at 16q24.2 locus 20

Supplementary Figure 7. Plot of effect size and mean age of cohorts with homogeneous phenotyping method for lead variant at 7p22.3 locus 21

Supplementary Figure 8. Plot of effect size and mean age of cohorts with homogeneous phenotyping method for lead variant at 12q23.3 locus 22

Supplementary Figure 9. Plot of effect size and mean age of cohorts with homogeneous phenotyping method for lead variant at 22q13.1 locus 23

Supplementary Figure 10. Plot of effect size and mean age of cohorts with homogeneous phenotyping method for lead variant at 11q23.1 locus 24

Supplementary Figure 11. Plots of Z-scores by cohort sample size for 7 lead variants 25

Supplementary Figure 12. PM-plots for 7 lead variants 27

Supplementary Figure 13A. Miami plot for sex-stratified GWA meta-analysis 30

Supplementary Figure 13B. Quantile-quantile plot for sex-stratified GWA meta-analysis 30

Supplementary Figure 14. Functional annotation of genome-wide significant variants from combined GWA meta-analysis (stage 3) 31

Supplementary Figure 15. The lead SNP at 22q13.1 (rs4820299) and its association with differential expression of *TRIOBP* gene in brain tissue 32

Supplementary Figure 16. Association between lateral ventricular volume and brain-specific gene-expression levels from GTEx project using MetaXcan method 33

Supplementary Figure 17. Distribution plot of untransformed and transformed lateral ventricular volume in different cohorts 34

Supplementary Table 1. Genomic inflation factor for the individual studies 43

Supplementary Table 2. Partitioned heritability analysis performed on lateral ventricular volume dataset 44

Supplementary Table 3. The results of gene-based analysis using VEGAS2 45

Supplementary Table 4. Phenotypic correlation between lateral ventricular volume and MRI phenotypes 46

Supplementary Table 5. Association of genetic risk score for neurological or psychiatric diseases with lateral ventricular volume 46

Supplementary Table 6. Tau SNPs used for genetic risk score analysis and their association with lateral ventricular volume 47

Supplementary References 48

Supplementary Note 1

Studies participating in GWA meta-analysis (stage 1)

Age, Gene/Environment Susceptibility-Reykjavik Study (AGES- Reykjavik)

The Reykjavik Study cohort originally comprised a random sample of 30,795 men and women born in 1907-1935 and living in Reykjavik in 1967.[1] A total of 19381 attended, resulting in 71% recruitment rate. The study sample was divided into six groups by birth year and birth date within a month. One group was designated for longitudinal follow-up and was examined in all stages. One group was designated a control group and was not included in examinations until 1991. Other groups were invited to participate in specific stages of the study. Between 2002 and 2006, the AGES-Reykjavik study re-examined 5764 survivors of the original cohort who had participated before in the Reykjavik Study. The study is approved by the Icelandic National Bioethics Committee, (VSN: 00–063), the Icelandic Data Protection Authority, and the MedStar Institutional Review Board. All subjects provided written informed consent.

The Austrian Stroke Prevention Study (ASPS)

The ASPS study is a single center prospective follow-up study on the effects of vascular risk factors on brain structure and function in the normal elderly population of the city of Graz, Austria. The procedure of recruitment and diagnostic work-up of study participants has been described previously.[2,3] A total of 2007 participants were randomly selected from the official community register stratified by gender and 5 year age groups. Individuals were excluded from the study if they had a history of neuropsychiatric disease, including previous stroke, transient ischemic attacks, and dementia, or an abnormal neurologic examination determined on the basis of a structured clinical interview and a physical and neurologic examination. During 2 study periods between September 1991 and March 1994 and between January 1999 and December 2003 an extended diagnostic work-up including MRI and neuropsychological testing was done in 1076 individuals aged 45 to 85 years randomly selected from the entire cohort: 509 from the first period and 567 from the second. In 1992, blood was drawn from all study participants for DNA extraction. They were all European Caucasians. Genotyping was performed in 996 participants, and the 768 who also underwent MRI scanning at baseline were available for these analyses. The study protocol was approved by the ethics committee of the Medical University of Graz (Austria), and written informed consent was obtained from all participants.

The Cardiovascular Health Study (CHS)

The CHS is a population-based cohort study of risk factors for coronary heart disease and stroke in adults ≥65 years conducted across four field centers.[4] The original predominantly European ancestry cohort of 5,201 persons was recruited in 1989-1990 from random samples of the Medicare eligibility lists; subsequently, an additional predominantly African-American cohort of 687 persons was enrolled for a total sample of 5,888. Blood samples were drawn from all participants at their baseline examination and DNA was subsequently extracted from available samples. Genotyping was performed at the General Clinical Research Center’s Phenotyping/Genotyping Laboratory at Cedars-Sinai among CHS participants who consented to genetic testing and had DNA. European ancestry participants were excluded from the GWAS study sample due to the presence at study baseline of coronary heart disease, congestive heart failure, peripheral vascular disease, valvular heart disease, stroke or transient ischemic attack or lack of available DNA. Among those with successful GWAS, 2155 European ancestry and 507 African-American participants had available MRI scans for analysis. CHS was approved by institutional review committees at each field center and individuals in the present analysis

had available DNA and gave informed consent including consent to use of genetic information for the study of cardiovascular disease.

Erasmus Rucphen Family study (ERF)

Erasmus Rucphen Family is a family-based cohort study that includes inhabitants of a genetically isolated community in the South-West of the Netherlands, studied as part of the Genetic Research in Isolated Population (GRIP) program. The goal of the study is to identify the risk factors in the development of complex disorders. The study population includes approximately 3,000 individuals who are living descendants of 22 couples who lived in the isolate between 1850 and 1900 and had at least six children baptized in the community church. All data were collected between 2002 and 2005. All participants gave informed consent, and the Medical Ethics Committee of the Erasmus University Medical Centre approved the study.

Lothian Birth Cohort 1936 (LBC1936)

The LBC1936 were all born in 1936 and participated in the Scottish Mental Survey 1947 (SMS1947) (Scottish Council for Research in Education Mental Survey Committee, 1949). They represent individuals living in the community in the Lothian region of Scotland.[5] Between 2008 and 2010, 866 (418 female) cohort members aged ~73 years participated in medical, genetic, and cognitive testing, including detailed brain MRI.[6] Ventricular volume measurements and genome-wide genotype data were available for 565 individuals (53.3% male) aged 72.7 years (SD = 0.73) years. All individuals reported being healthy: none reported cognitive pathologies and a brief dementia screening test showed Mini-Mental State Examination (MMSE) scores were ≥ 24. Ethical approval for the study was via the Lothian (REC 07/MRE00/58) and Scottish Multicenter (MREC/01/0/56) Research Ethics Committees, with written informed consent obtained from all participants.

Pravastatin in the Elderly at Risk (PROSPER)

PROSPER was a prospective multicenter randomized placebo-controlled trial to assess whether treatment with pravastatin diminishes the risk of major vascular events in elderly.[7, 8, 9] Between December 1997 and May 1999, we screened and enrolled subjects in Scotland (Glasgow), Ireland (Cork), and the Netherlands (Leiden). Men and women aged 70-82 years were recruited if they had pre-existing vascular disease or increased risk of such disease because of smoking, hypertension, or diabetes. A total number of 5,804 subjects were randomly assigned to pravastatin or placebo. A large number of prospective tests were performed including Biobank tests and cognitive function measurements. The PROSPER study was approved by the institutional ethics review boards of centers of Cork University (Ireland), Glasgow University (Scotland) and Leiden University Medical Center (the Netherlands) and all participants gave written informed consent. Our institutional ethics review board approved the protocol for the MRI substudy, and all participants gave written informed consent.

Rotterdam Study (RS)

The Rotterdam Study is a prospective, population-based cohort study among individuals living in the well-defined Ommoord district in the city of Rotterdam in The Netherlands. The aim of the study is to determine the occurrence of cardiovascular, neurological, ophthalmic, endocrine, hepatic, respiratory, and psychiatric diseases in elderly people. The cohort was initially defined in 1990 among approximately 7,900 persons, aged 55 years and older, who underwent a home interview and extensive physical examination at the baseline and during follow-up rounds every 3-4 years (RS-I). The cohort was extended in 2000/2001 (RS-II, 3,011 individuals aged 55 years and older) and 2006/2008 (RS-III,

3,932 subjects, aged 45 and older). Written informed consent was obtained from all participants and the Medical Ethics Committee of the Erasmus Medical Center, Rotterdam, approved the study.

RUSH-ROSMAP

The Rush Memory and Aging Project (MAP), started in 1997, enrolled older men and women from assisted living facilities in the Chicagoland area with no evidence on dementia at baseline. All participants agreed to annual clinical evaluations and signed both an informed consent and an Anatomic Gift Act donating their brains, spinal cords and selected nerves and muscles to Rush investigators at the time of death. An imaging substudy was initiated in 2008.[10]

The Religious Order Study (ROS), started in 1994, enrolled Catholic priests, nuns, and brothers, aged 53 or older from about 40 groups in 12 states. All participants were free of known dementia at enrollment, agreed to annual clinical evaluations and signed both an informed consent and an Anatomic Gift Act donating their brains at the time of death. An imaging substudy was initiated in 2010.[11] ROS, MAP, and the imaging studies were approved by the Institutional Review Board of Rush University Medical Center.

Studies participating in GWAS meta-analysis (stage 2)

Atherosclerosis Risk in Communities (ARIC)

The ARIC study is a population-based cohort study of atherosclerosis and clinical atherosclerotic diseases.[12] At its inception (1987-1989), 15,792 men and women, including 11,478 white and 4,266 black participants were recruited from four U.S. communities: Suburban Minneapolis, Minnesota; Washington County, Maryland; Forsyth County, North Carolina; and Jackson, Mississippi. In the first 3 communities, the sample reflects the demographic composition of the community. In Jackson, only black residents were enrolled. Participants were between age 45 and 64 years at their baseline examination in 1987-1989 when blood was drawn for DNA extraction and participants consented to genetic testing. Vascular risk factors and outcomes, including transient ischemic attack, stroke, and dementia, were determined in a standard fashion. During the first 2 years (1993-1994) of the third ARIC examination (V3), participants aged 55 and older from the Forsyth County and Jackson sites were invited to undergo cranial MRI. This subgroup of individuals with MRI scanning represents a random sample of the full cohort because examination dates were allocated at baseline through randomly selected induction cycles. Between 2011 and 2013, ARIC conducted a fifth examination (V5), during which MRI scan was also conducted. For the V5 sample in this meta-analysis, we excluded individuals who had been included in the V3 sample. The Institutional Review Board at each participating institution approved the ARIC study and all participants provided informed consent before each examination.

Coronary Artery Risk Development in Young Adults (CARDIA)

The CARDIA study is a population-based, prospective cohort examining the development and determinants of clinical and subclinical cardiovascular disease and its risk factors.[13] The CARDIA study initial enrollment consisted of 5115 European Americans and African American men and women between 18 and 30 years old (52% African American and 55% women). The study is multicenter with recruitment in Birmingham, AL; Chicago, IL; Minneapolis, MN; and Oakland, CA. Baseline measurements were repeated, and additional measurements performed, at Years 2, 5, 7, 10, 15, 20, and 25. The CARDIA study was approved by the institutional review boards of the

coordinating center and the four participating field centers, and written informed consent was obtained from participants at all examinations.

Framingham Heart Study (FHS)

The FHS is a three-generation, single-site, community-based, ongoing cohort study initiated in 1948 to investigate the risk factors for cardiovascular disease.[14, 15] It now comprises 3 generations of participants: the Original cohort followed since 1948; their Offspring and spouses of the Offspring (Gen 2), followed since 1971; and children from the largest Offspring families enrolled in 2000 (Gen 3).[16] The Original cohort enrolled 5,209 men and women who comprised two-thirds of the adult population then residing in Framingham, MA. Survivors continue to receive biennial examinations. The Offspring cohort comprises 5,124 persons (including 3,514 biological offspring) who have been examined approximately once every 4 years. The Third-generation includes 4,095 participants with at least one parent in the Offspring Cohort. The first two generations were invited to undergo an initial brain MRI in 1999-2005, and for Gen 3, brain MRI began in 2009. The population of Framingham was virtually entirely white (Europeans of English, Scots, Irish and Italian descent) in 1948 when the Original cohort was recruited. Self-reports of ethnicity across all three generations were 99.7% whites, reflecting the ethnicity of the population of Framingham in 1948. All participants provided written informed consent at each examination. Study protocols and consent forms were approved by the Institutional Review Board of the Boston University Medical Center.

LIFE-Adult

LIFE-Adult is a population-based study of 10,000 inhabitants (18 - 79 years) of the city of Leipzig (Saxony, Germany).[17] Individuals were phenotyped for several health and disease related parameters. All subjects gave written informed consent to participate in the study. The study was conducted according to the Declaration of Helsinki and approved by the ethics committee of the University of Leipzig (registration-number: 263-2009-14122009).

Sydney Memory and Aging Study (MAS)

Participants were randomly recruited from the compulsory electoral rolls of Sydney. Participants were aged 70-90 years old and free of dementia and psychotic symptoms at baseline. Participants provided written informed consent and ethics approval was granted from the University of New South Wales and the Illawarra Area Health Service Human Research Ethics Committees. For further details of the study see Sachdev et al., 2010.[18]

Older Australian Twins Study (OATS)

Participants aged 65 years and over were recruited from the Australian Twin Registry and from other avenues. Exclusion criteria included insufficient English to complete the assessment. All participants provided written informed consent and approval was granted from the Australian Twin Registry, University of New South Wales, University of Melbourne, Queensland Institute of Medical Research and the South Eastern Sydney and Illawarra Area Health Service. For further details see Sachdev et al., 2009.[19]

Saguenay Youth Study (SYS)

The Saguenay Youth Study is a two-generational study of adolescents and their parents (n = 1029 adolescents and 962 parents) aimed at investigating the aetiology, early stages and trans-generational trajectories of common cardiometabolic and brain diseases.[20] The cohort was recruited from the

genetic founder population of the Saguenay Lac St Jean region of Quebec, Canada. The participants underwent extensive (15-h) phenotyping. The data collection took place during 2003-12 in adolescents (full) and their parents (partial), and during 2012-15 in parents (full).[20] The Research Ethics Committee of the Chicoutimi Hospital approved the study protocol. Written informed consent was also obtained from all the participants.

Study of Health in Pomerania (SHIP)

The Study of Health in Pomerania is a population-based project in West Pomerania, the north-east area of Germany.[21, 22] A sample from the population aged 20 to 79 years was drawn from population registries. First, the three cities of the region (with 17,076 to 65,977 inhabitants) and the 12 towns (with 1,516 to 3,044 inhabitants) were selected, and then 17 out of 97 smaller towns (with less than 1,500 inhabitants), were drawn at random. Second, from each of the selected communities, subjects were drawn at random, proportional to the population size of each community and stratified by age and gender. Only individuals with German citizenship and main residency in the study area were included. Finally, 7,008 subjects were sampled, with 292 persons of each gender in each of the twelve five-year age strata. In order to minimize drop-outs by migration or death, subjects were selected in two waves. The net sample (without migrated or deceased persons) comprised 6,267 eligible subjects. Selected persons received a maximum of three written invitations. In the case of non-response, letters were followed by a phone call or by home visits if contact by phone was not possible. The SHIP population finally comprised 4,308 participants (corresponding to a final response of 68.8%). Follow-up examination (SHIP-1) was conducted 5 years after baseline (2002-2006) and included 3300 subjects. From 2008 to 2012 the third phase of data collection (SHIP-2, N=2333) was carried out including a whole body MRI. SHIP was fully approved by the ethics board of the University Medicine Greifswald. All participants have given written informed consent.

Study of Health in Pomerania (SHIP-Trend)

The Study of Health in Pomerania (SHIP-Trend) is a population-based cohort study in West Pomerania, a region in the northeast of Germany, assessing the prevalence and incidence of common population-relevant diseases and their risk factors.[22] Baseline examinations for SHIP-Trend were carried out between 2008 and 2012, comprising 4,420 participants aged 20 to 81 years. Study design and sampling methods were previously described. The medical ethics committee of the University of Greifswald approved the study protocol, and oral and written informed consents were obtained from each of the study participants. The baseline examinations (SHIP-TREND-0) were performed from June 2009 until October 2012 (n=4422) and included a whole body MRI. SHIP-TREND was fully approved by the ethics board of the University Medicine Greifswald. All participants have given written informed consent.

The Vietnam Era Twin Study of Aging (VETSA)

The Vietnam Era Twin Study of Aging (VETSA) is a longitudinal behavior genetics study of cognitive and brain aging.[23] Participants are members of the Vietnam Era Twin Registry, which is housed at the VA Puget Sound Health Care System in Seattle, WA, USA.[24] All of the twins served in some branch of US military service at some time during the Vietnam era (1965-1975). VETSA participants live throughout the United States. The sample is primarily Caucasian (European-American): 86% based on self-report. The average educational attainment is 13.8 years (SD=2.1). At wave 1, 79% were married, and 78% were employed full-time. Nearly 80% report no combat experience. The sample is similar with respect to health and lifestyle characteristics to American men in their age range based on US Center for Disease Control and Prevention data. VETSA is currently in

its third wave of data collection. Data for this report were from wave 1. The study is approved by the Institutional Review Boards at all of the participating institutions, and all participants gave written informed consent to participate. In addition, participants included in this report gave written consent for their data, including DNA data, to be used in other research studies.

Generation R

Generation R is a population-based birth cohort aiming to identify early environmental and genetic determinants of development and health.[25] All parents gave informed consent for their children's participation. The Generation R Study is conducted in accordance with the World Medical Association Declaration of Helsinki and study protocols have been approved by the Medical Ethics Committee of the Erasmus Medical Center, Rotterdam.

Supplementary Note 2

Funding sources

Age, Gene/Environment Susceptibility-Reykjavik Study (AGES - Reykjavik): This study has been funded by NIH contracts N01-AG-1-2100 and 271201200022C, the NIA Intramural Research Program, Hjartavernd (the Icelandic Heart Association), and the Althingi (the Icelandic Parliament). The study is approved by the Icelandic National Bioethics Committee, VSN: 00-063. The researchers are indebted to the participants for their willingness to participate in the study.

The Austrian Stroke Prevention Study (ASPS): The authors thank the staff and the participants for their valuable contributions. We thank Birgit Reinhart for her long-term administrative commitment, Elfi Hofer for the technical assistance at creating the DNA bank, Ing. Johann Semmler and Anita Harb for DNA sequencing and DNA analyses by TaqMan assays and Irmgard Poelzl for supervising the quality management processes after ISO9001 at the biobanking and DNA analyses. The research reported in this article was funded by the Austrian Science Fond (FWF) grant number P20545-P05 and P13180 and supported by the Austrian National Bank Anniversary Fund, P15435, the Austrian Ministry of Science under the aegis of the EU Joint Programme-Neurodegenerative Disease Research (JPND)-www.jpnd.eu and by the Austrian Science Fund P20545-B05. The Medical University of Graz supports the databank of the ASPS.

Cardiovascular Health Study (CHS): This CHS research was supported by NHLBI contracts HHSN268201200036C, HHSN268200800007C, N01HC55222, N01HC85079, N01HC85080, N01HC85081, N01HC85082, N01HC85083, N01HC85086, N01HC15103, and HHSN268200960009C; and NHLBI grants U01HL080295, R01HL087652, R01HL105756, R01HL103612, R01HL120393, and R01HL130114 with additional contribution from the National Institute of Neurological Disorders and Stroke (NINDS). Additional support was provided through R01AG023629, N01HC15103, and R01AG20098 from the National Institute on Aging (NIA). A full list of principal CHS investigators and institutions can be found at CHS-NHLBI.org. The provision of genotyping data was supported in part by the National Center for Advancing Translational Sciences, CTSI grant UL1TR000124, and the National Institute of Diabetes and Digestive and Kidney Disease Diabetes Research Center (DRC) grant DK063491 to the Southern California Diabetes Endocrinology Research Center. The content is solely the responsibility of the authors and does not necessarily represent the official views of the National Institutes of Health.

Erasmus Rucphen Family study (ERF): The Erasmus Rucphen Family study (ERF) study as a part of EUROSPAN (European Special Populations Research Network) was supported by European Commission FP6 STRP grant number 018947 (LSHG-CT-2006-01947) and also received funding from the European Community's Seventh Framework Programme (FP7/2007-2013)/grant agreement HEALTH-F4-2007-201413 by the European Commission under the programme "Quality of Life and Management of the Living Resources" of 5th Framework Programme (no. QLG2-CT-2002-01254). High-throughput analysis of the ERF data was supported by joint grant from Netherlands Organization for Scientific Research and the Russian Foundation for Basic Research (NWO-RFBR 047.017.043). Exome sequencing analysis in ERF was supported by the ZonMw grant (project 91111025). This work has been performed as part of the CoSTREAM project (www.costream.eu) and has received funding from the European Union's Horizon 2020 research and innovation programme under grant agreement No 667375. We are grateful to all study participants and their relatives, general practitioners and neurologists for their contributions and to P. Veraart for her help in genealogy, J. Vergeer for the

supervision of the laboratory work and P. Snijders for his help in data collection. Najaf Amin is supported by the Netherlands Brain Foundation (project number F2013(1)-28).

Lothian Birth Cohort 1936 (LBC1936): The work was undertaken as part of the Cross Council and University of Edinburgh Centre for Cognitive Ageing and Cognitive Epidemiology (CCACE; http://www.ccace.ed.ac.uk). This work was supported by a Research into Ageing programme grant (to I.J.D.) and the Age UK-funded Disconnected Mind project (http://www.disconnectedmind.ed.ac.uk; to I.J.D. and J.M.W.), with additional funding from the UK Medical Research Council (MRC Mr/M01311/1, G1001245/96077, G0701120/79365 to I.J.D., J.M.W. and M.E.B.). The whole genome association part of this study was funded by the Biotechnology and Biological Sciences Research Council (BBSRC; Ref. BB/F019394/1). J.M.W. is supported by the Scottish Funding Council through the SINAPSE Collaboration (http://www.sinapse.ac.uk). CCACE (MRC MR/K026992/1) is funded by the BBSRC and MRC. The image acquisition and analysis were performed at the Brain Research Imaging Centre, University of Edinburgh (http://www.bric.ed.ac.uk). MVH is supported by the Row Fogo Charitable Trust.

Pravastatin in the Elderly at Risk (PROSPER): The PROSPER study was supported by an investigator initiated grant obtained from Bristol-Myers Squibb. Prof. Dr. J. W. Jukema is an Established Clinical Investigator of the Netherlands Heart Foundation (grant 2001 D 032). Support for genotyping was provided by the seventh framework program of the European commission (grant 223004) and by the Netherlands Genomics Initiative (Netherlands Consortium for Healthy Aging grant 050-060-810).

Rotterdam Study (RS): The generation and management of GWAS genotype data for the Rotterdam Study (RS I, RS II, RS III) were executed by the Human Genotyping Facility of the Genetic Laboratory of the Department of Internal Medicine, Erasmus MC, Rotterdam, The Netherlands. The GWAS datasets are supported by the Netherlands Organisation of Scientific Research NWO Investments (nr. 175.010.2005.011, 911-03-012), the Genetic Laboratory of the Department of Internal Medicine, Erasmus MC, the Research Institute for Diseases in the Elderly (014-93-015; RIDE2), the Netherlands Genomics Initiative (NGI)/Netherlands Organisation for Scientific Research (NWO) Netherlands Consortium for Healthy Aging (NCHA), project nr. 050-060-810. This work has been performed as part of the CoSTREAM project (www.costream.eu) and has received funding from the European Union's Horizon 2020 research and innovation programme under grant agreement No 667375. This project has also received funding from the European Research Council (ERC) under the European Union’s Horizon 2020 research and innovation programme (project: ORACLE, grant agreement No: 678543).

We thank Pascal Arp, Mila Jhamai, Marijn Verkerk, Lizbeth Herrera and Marjolein Peters, and Carolina Medina-Gomez, for their help in creating the GWAS database, and Karol Estrada, Yurii Aulchenko, and Carolina Medina-Gomez, for the creation and analysis of imputed data. The Rotterdam Study is funded by Erasmus Medical Center and Erasmus University, Rotterdam, Netherlands Organization for the Health Research and Development (ZonMw), the Research Institute for Diseases in the Elderly (RIDE), the Ministry of Education, Culture and Science, the Ministry for Health, Welfare and Sports, the European Commission (DG XII), and the Municipality of Rotterdam. The authors are grateful to the study participants, the staff from the Rotterdam Study and the participating general practitioners and pharmacists.

RUSH-ROSMAP: The RUSH-ROSMAP is funded by National Institute on Aging grants R01AG17917, R01AG15819, and P30AG10161, and the Illinois Department of Public Health. We

thank all the participants of ROS and MAP and the investigators and staff at the Rush Alzheimer's Disease Center.

Atherosclerosis Risk in Communities (ARIC): The Atherosclerosis Risk in Communities Study is carried out as a collaborative study supported by National Heart, Lung, and Blood Institute contracts (HHSN268201100005C, HHSN268201100006C, HHSN268201100007C, HHSN268201100008C, HHSN268201100009C, HHSN268201100010C, HHSN268201100011C, and HHSN268201100012C), R01HL087641, R01HL59367 and R01HL086694; National Human Genome Research Institute contract U01HG004402; and National Institutes of Health contract HHSN268200625226C. The authors thank the staff and participants of the ARIC study for their important contributions. Infrastructure was partly supported by Grant Number UL1RR025005, a component of the National Institutes of Health and NIH Roadmap for Medical Research.

Coronary Artery Risk Development in Young Adults (CARDIA): The CARDIA Study is conducted and supported by the National Heart, Lung, and Blood Institute in collaboration with the University of Alabama at Birmingham (HHSN268201300025C & HHSN268201300026C), Northwestern University (HHSN268201300027C), University of Minnesota (HHSN268201300028C), Kaiser Foundation Research Institute (HHSN268201300029C), and Johns Hopkins University School of Medicine (HHSN268200900041C). CARDIA is also partially supported by the Intramural Research Program of the National Institute on Aging. Genotyping was funded as part of the NHLBI Candidate-gene Association Resource (N01-HC-65226) and the NHGRI Gene Environment Association Studies (GENEVA) (U01-HG004729, U01-HG04424, and U01-HG004446). This work was also partially supported by NIH grant NS087541 to MF. This manuscript has been reviewed and approved by CARDIA for scientific content.

Framingham Heart Study (FHS): The Framingham Heart Study is supported by the National Heart, Lung and Blood Institute (NHLBI) Framingham Heart Study (contracts N01-HC-25195 and HHSN268201500001I), the Boston University School of Medicine, and by grants from the National Institute of Health (AG054076, AG033193, AG049505, AG033040, NS017950, AG049607, AG010129). We would like to thank the dedication of the Framingham Study participant. We also thank the Framingham study team, especially the Neurology core, both investigators and staff, for their contributions to data collection.

LIFE-Adult: The LIFE-Adult was funded by the Leipzig Research Center for Civilization Diseases (LIFE). LIFE is an organizational unit affiliated to the Medical Faculty of the University of Leipzig. LIFE is funded by means of the European Union, by the European Regional Development Fund (ERDF) and by funds of the Free State of Saxony within the framework of the excellence initiative (Grant Numbers: 713-241202, 713-241202, 14505/2470, 14575/2470). Analysis was also funded by the Deutsche Forschungsgemeinschaft (Grant Number: CRC 1052 “Obesity mechanisms” A1). We thank the participants of LIFE-Adult very much for their time and blood samples. We thank Kerstin Wirkner very much for running the LIFE study center and Ralph Burkhardt for genotyping.

Sydney Memory and Aging Study (Sydney MAS): The Sydney MAS study is funded by National and Health Medical Research Council (NHMRC) Program and Project Grants (ID350833, ID568969, ID109308). We would like to acknowledge and gratefully thank the participants and their supporters. We also thank the Sydney MAS Research Team.

Older Australian Twins Study (OATS): The OATS study is funded by NHMRC Program Grant ID1045325 and the NHMRC/Australian Research Council Strategic Award (ID401162). The

Australian Twin Registry is supported by the NHRMC Enabling Grant 310667. We would like to acknowledge and gratefully thank the participants and their supporters. We also thank the OATS Research Team.

Saguenay Youth Study (SYS): The Canadian Institutes of Health Research and the Heart and Stroke Foundation of Canada fund the SYS. TP is the Tanenbaum Chair in Population Neuroscience at the Rotman Research Institute, University of Toronto and the Dr. John and Consuela Phelan Scholar at Child Mind Institute, New York. Computations were performed on the GPC supercomputer at the SciNet HPC Consortium. SciNet is funded by: the Canada Foundation for Innovation under the auspices of Compute Canada; the Government of Ontario; Ontario Research Fund - Research Excellence; and the University of Toronto. The funders had no role in study design, data collection, and analysis, decision to publish, or preparation of the manuscript.

Study of Health in Pomerania (SHIP and SHIP-Trend): SHIP is part of the Community Medicine Research net of the University of Greifswald, Germany, which is funded by the Federal Ministry of Education and Research (grants no. 01ZZ9603, 01ZZ0103, and 01ZZ0403), the Ministry of Cultural Affairs as well as the Social Ministry of the Federal State of Mecklenburg-West Pomerania, and the network 'Greifswald Approach to Individualized Medicine (GANI_MED)' funded by the Federal Ministry of Education and Research (grant 03IS2061A). Genome-wide data have been supported by the Federal Ministry of Education and Research (grant no. 03ZIK012) and a joint grant from Siemens Healthineers, Erlangen, Germany and the Federal State of Mecklenburg- West Pomerania. MRI scans in SHIP and SHIP-TREND have been supported by a joint grant from Siemens Healthineers, Erlangen, Germany and the Federal State of Mecklenburg-West Pomerania. The University of Greifswald is a member of the Caché Campus program of the InterSystems GmbH.

Vietnam Era Twin Study of Aging (VETSA): The VETSA is funded by the US National Institute on Aging (grants no. NIH/NIA R01 AG050595, NIH/NIA R01 AG022381, NIH/NIA R01 AG054509, NIH/NIA R01 AG054002). SNP genotype for VETSA was performed by deCODE Genetics, Reykjavik, Iceland.

Generation R: The Generation R Study is conducted by the Erasmus Medical Center in close collaboration with the Erasmus University Rotterdam, Faculty of Social Sciences, the Municipal Health Service Rotterdam area, and the Stichting Trombosedienst and Artsenlaboratorium Rijnmond (STAR), Rotterdam. We gratefully acknowledge the contribution of general practitioners, hospitals, midwives and pharmacies in Rotterdam. The Generation R Study is made possible by financial support from Erasmus Medical Center, Rotterdam, and the Netherlands Organization for Health Research and Development (ZonMw). Supercomputing resources for MRI data were supported by the NWO Physical Sciences Division (Exacte Wetenschappen) and SURFsara (Cartesius HPC, project 15448). We would like to thank Anis Abuseiris, Karol Estrada, Dr. Tobias A. Knoch, and Rob de Graaf as well as their institutions Biophysical Genomics, Erasmus MC Rotterdam, The Netherlands, and especially the national German MediGRID and Services@MediGRID part of the German D-Grid, both funded by the German Bundesministerium fuer Forschung und Technology under grants #01 AK 803 A-H and # 01 IG 07015 G for access to their grid resources. A. Neumann and H. Tiemeier are supported by a grant of the Dutch Ministry of Education, Culture, and Science and the Netherlands Organization for Scientific Research (NWO grant No. 024.001.003, Consortium on Individual Development). The work of H. Tiemeier is further supported by a European Union's Horizon 2020 research and innovation program (Contract grant number: 633595, DynaHealth) and a NWO-VICI grant (NWO-ZonMW: 016.VICI.170.200). T. White is supported by the Netherlands Organization for

Health Research and Development (ZonMw) TOP project number 91211021. Supercomputing computations were supported by the NWO Physical Sciences Division (Exacte Wetenschappen) and SURFsara (Cartesius cluster, www.surfsara.nl).

Supplementary Figures

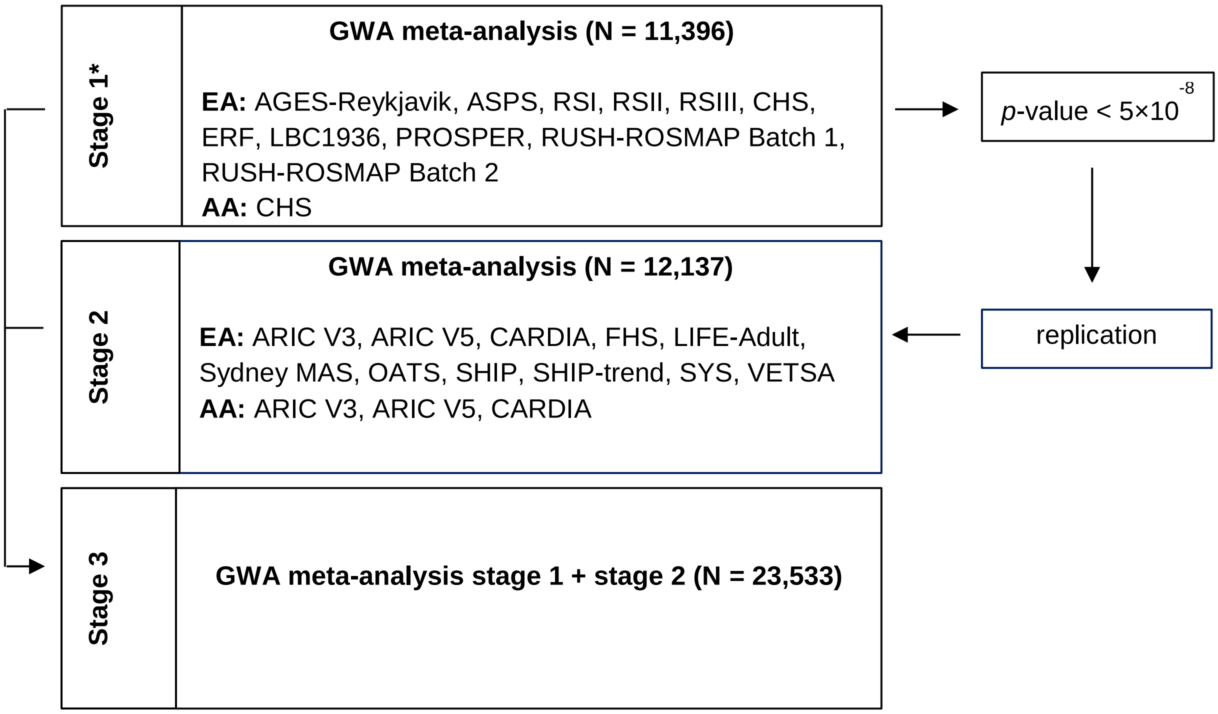

Supplementary Figure 1

An overview of study design.

Abbreviations: GWA - genome-wide association; N - sample size; EA - European ancestry; AA - African-American ancestry;
* Studies that uploaded summary statistic data before a certain deadline were included in stage 1. The deadline was set prior to data inspection and was not influenced by the results of the analyses.

A.

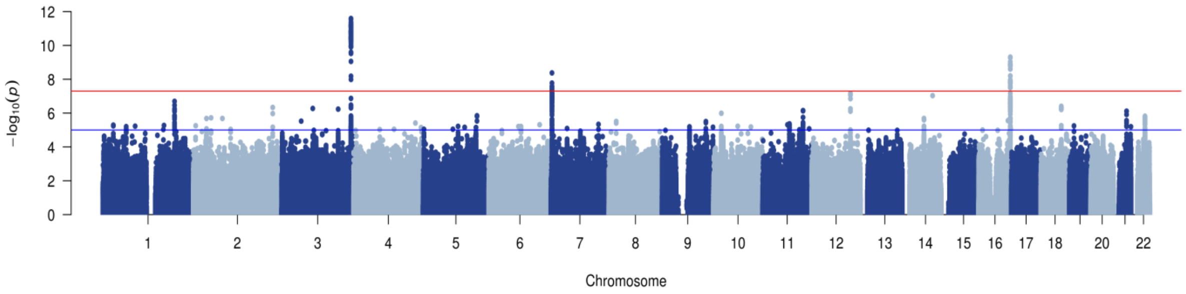

B.

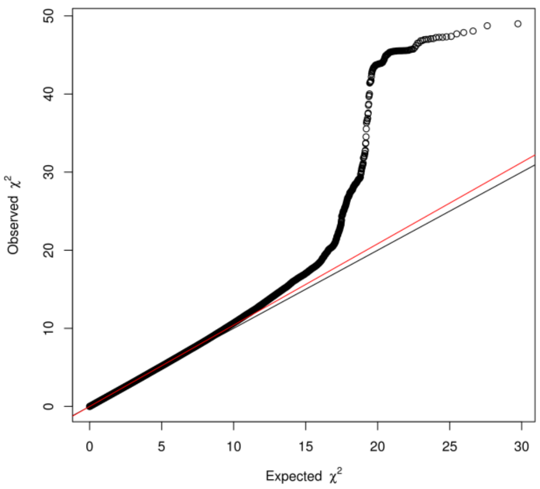

Supplementary Figure 2

A. Manhattan plot for stage 1 genome-wide association (GWA) meta-analysis. Each dot represents a variant. The plot shows –log10 *p*-values for all variants. Red line represents the genome-wide significance threshold (*p*-value < 5×10^{-8}), whereas blue line denotes suggestive threshold (*p*-value < 1×10^{-5}).

B. Quantile-quantile plot for stage 1 GWA meta-analysis.

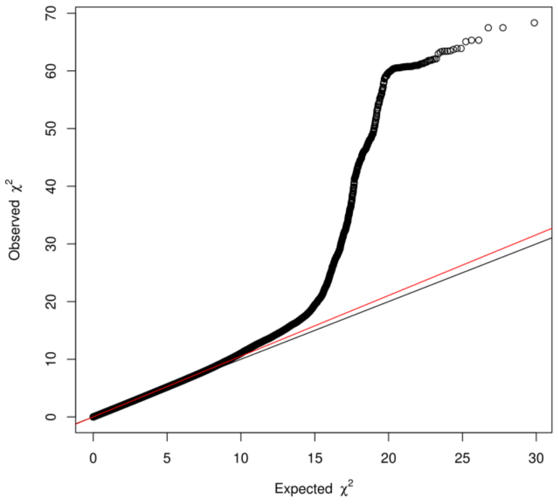

Supplementary Figure 3

Quantile-quantile plot for stage 3 GWA meta-analysis.

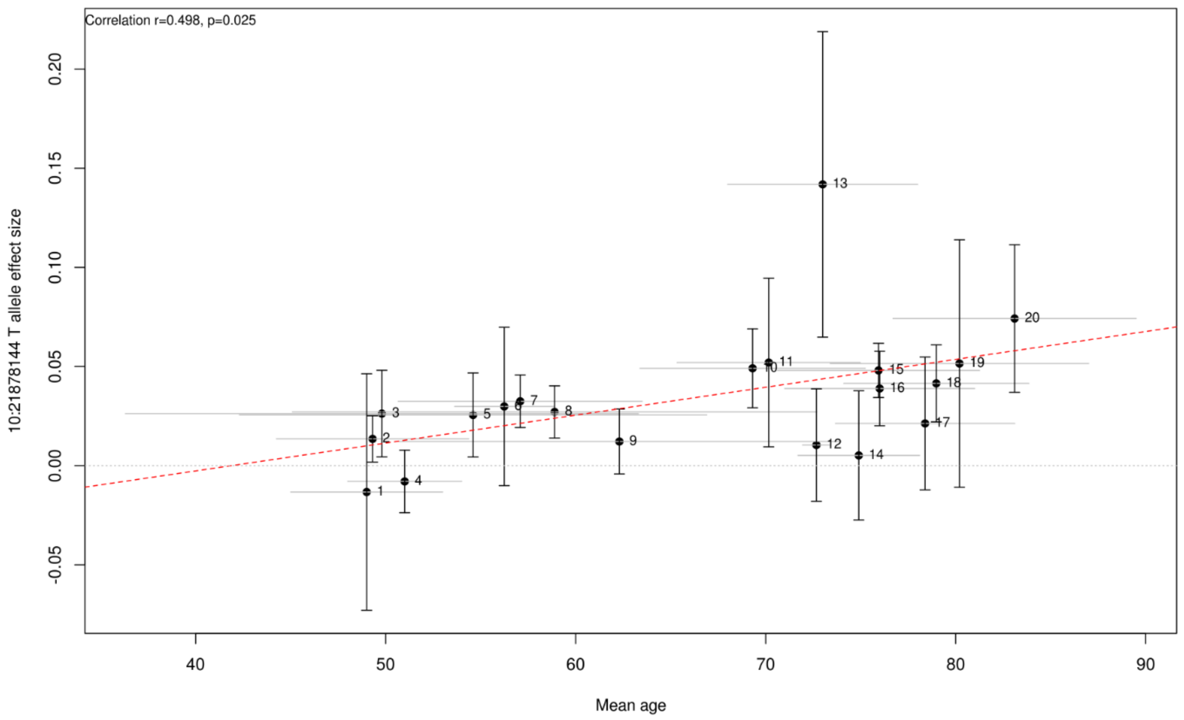

Supplementary Figure 4

Plot of effect size and mean age of cohorts with homogeneous phenotyping method for lead variant at 10p12.31 locus. Each number point denotes a cohort (1- CARDIA-AA, 2-SYS, 3-SHIP-trend, 4-CARDIA-EA, 5-SHIP, 6-VETSA, 7-RSIII, 8-FHS, 9-LIFE-Adult, 10-RSII, 11-OATS, 12-LBC1936, 13-ARIC-V5-AA, 14-PROSPER, 15-AGES, 16-ARIC-V5-EA, 17-MAS, 18-RSI, 19- RUSH-ROSMAP Batch 2, 20-RUSH-ROSMAP Batch 1). The plot includes SD of age range for each cohort.

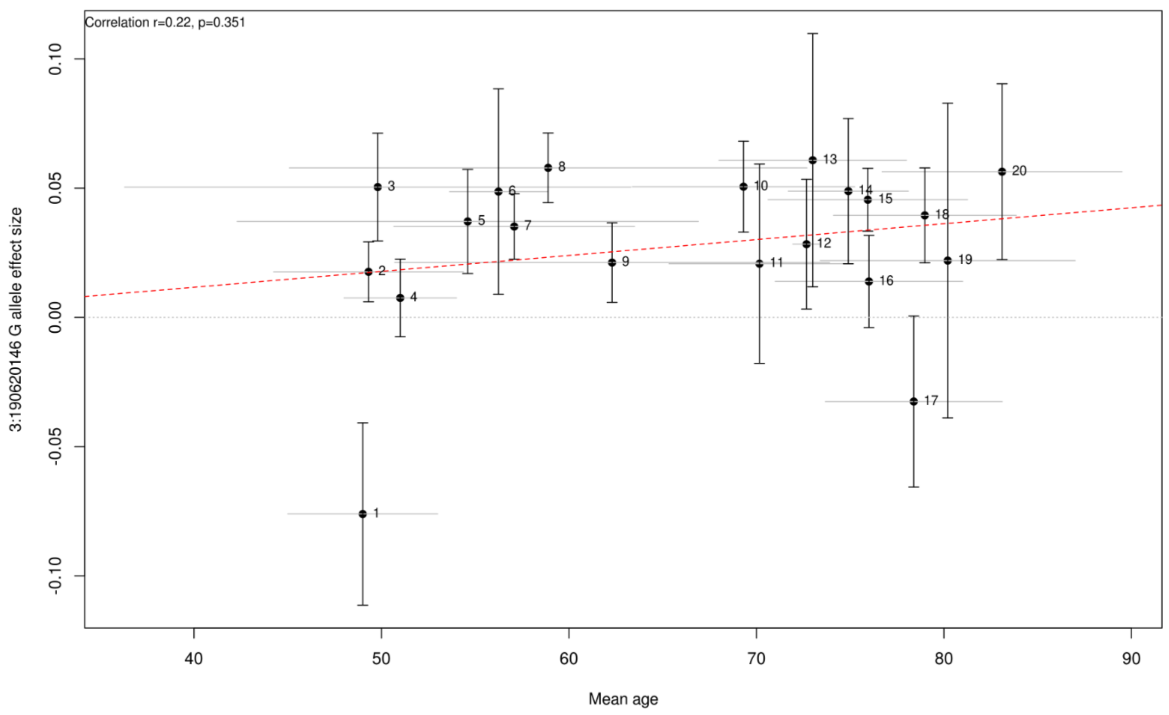

Supplementary Figure 5

Plot of effect size and mean age of cohorts with homogeneous phenotyping method for lead variant at 3q28 locus. Each number point denotes a cohort (1- CARDIA-AA, 2-SYS, 3-SHIP-trend, 4-CARDIA-EA, 5-SHIP, 6-VETSA, 7-RSIII, 8-FHS, 9-LIFE-Adult, 10-RSII, 11-OATS, 12-LBC1936, 13-ARIC-V5-AA, 14-PROSPER, 15-AGES, 16-ARIC-V5-EA, 17-MAS, 18-RSI, 19- RUSH-ROSMAP Batch 2, 20-RUSH-ROSMAP Batch 1). The plot includes SD of age range for each cohort.

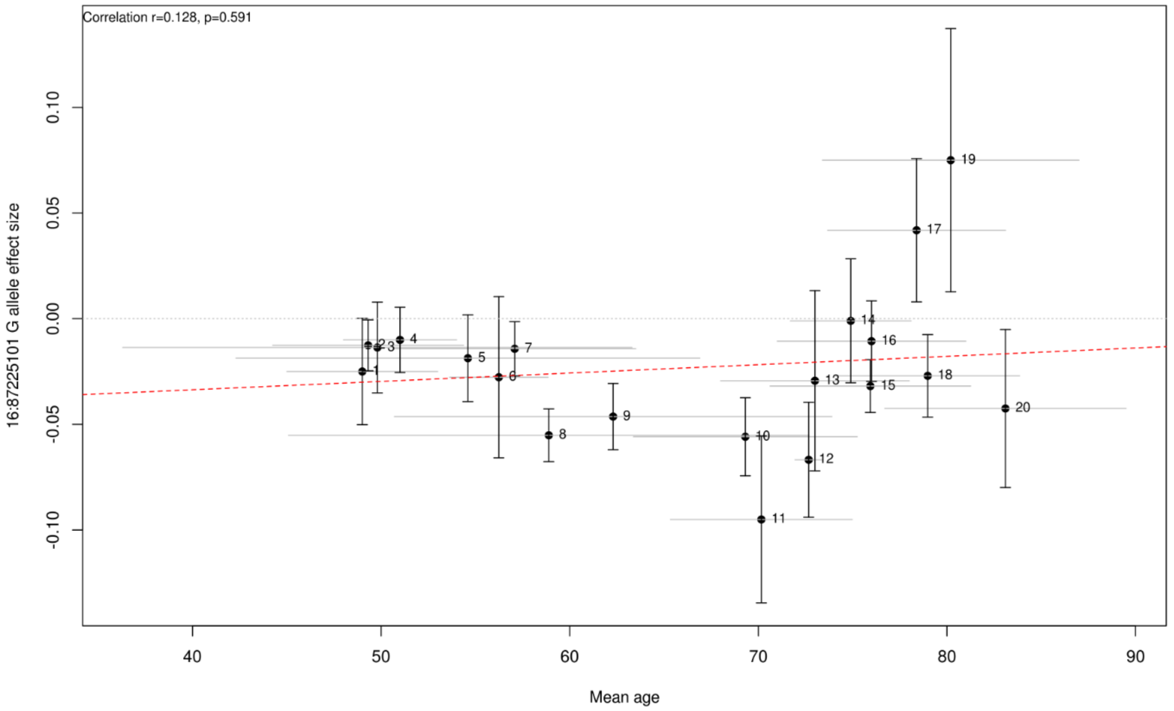

Supplementary Figure 6

Plot of effect size and mean age of cohorts with homogeneous phenotyping method for lead variant at 16q24.2 locus. Each number point denotes a cohort (1- CARDIA-AA, 2-SYS, 3-SHIP-trend, 4-CARDIA-EA, 5-SHIP, 6-VETSA, 7-RSIII, 8-FHS, 9-LIFE-Adult, 10-RSII, 11-OATS, 12-LBC1936, 13-ARIC-V5-AA, 14-PROSPER, 15-AGES, 16-ARIC-V5-EA, 17-MAS, 18-RSI, 19- RUSH-ROSMAP Batch 2, 20-RUSH-ROSMAP Batch 1). The plot includes SD of age range for each cohort.

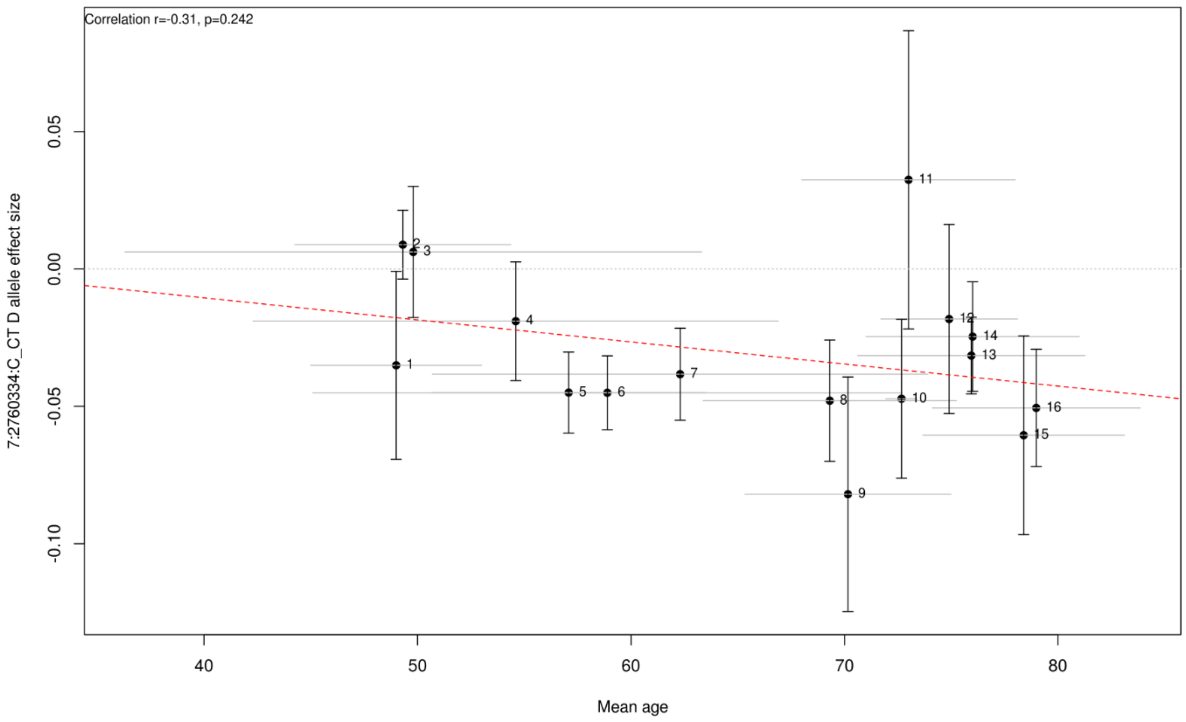

Supplementary Figure 7

Plot of effect size and mean age of cohorts with homogeneous phenotyping method for lead variant at 7p22.3 locus. Each number point denotes a cohort (1- CARDIA-AA, 2-SYS, 3-SHIP-trend, 4-SHIP, 5-RSIII, 6-FHS, 7-LIFE-Adult, 8-RSII, 9-OATS, 10-LBC1936, 11-ARIC-V5-AA, 12-PROSPER, 13-AGES, 14-ARIC-V5-EA, 15-MAS, 16-RSI). The plot includes SD of age range for each cohort.

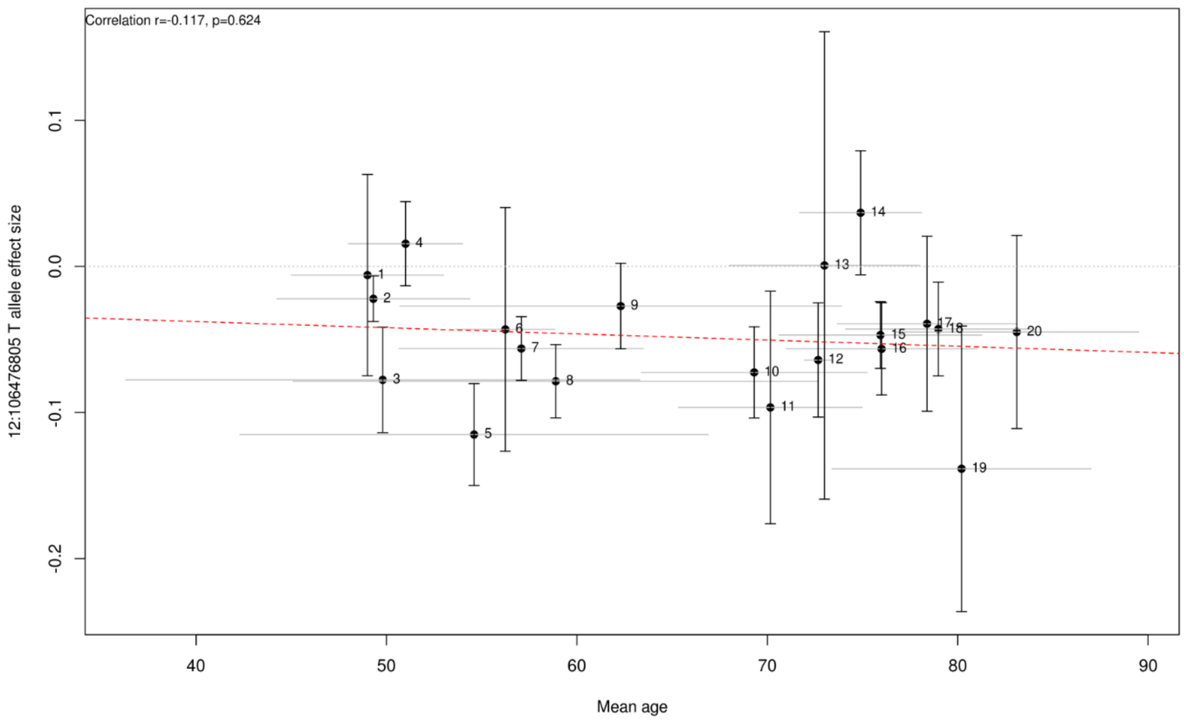

Supplementary Figure 8

Plot of effect size and mean age of cohorts with homogeneous phenotyping method for lead variant at 12q23.3 locus. Each number point denotes a cohort (1- CARDIA-AA, 2-SYS, 3-SHIP-trend, 4-CARDIA-EA, 5-SHIP, 6-VETSA, 7-RSIII, 8-FHS, 9-LIFE-Adult, 10-RSII, 11-OATS, 12-LBC1936, 13-ARIC-V5-AA, 14-PROSPER, 15-AGES, 16-ARIC-V5-EA, 17-MAS, 18-RSI, 19- RUSH-ROSMAP Batch 2, 20-RUSH-ROSMAP Batch 1). The plot includes SD of age range for each cohort.

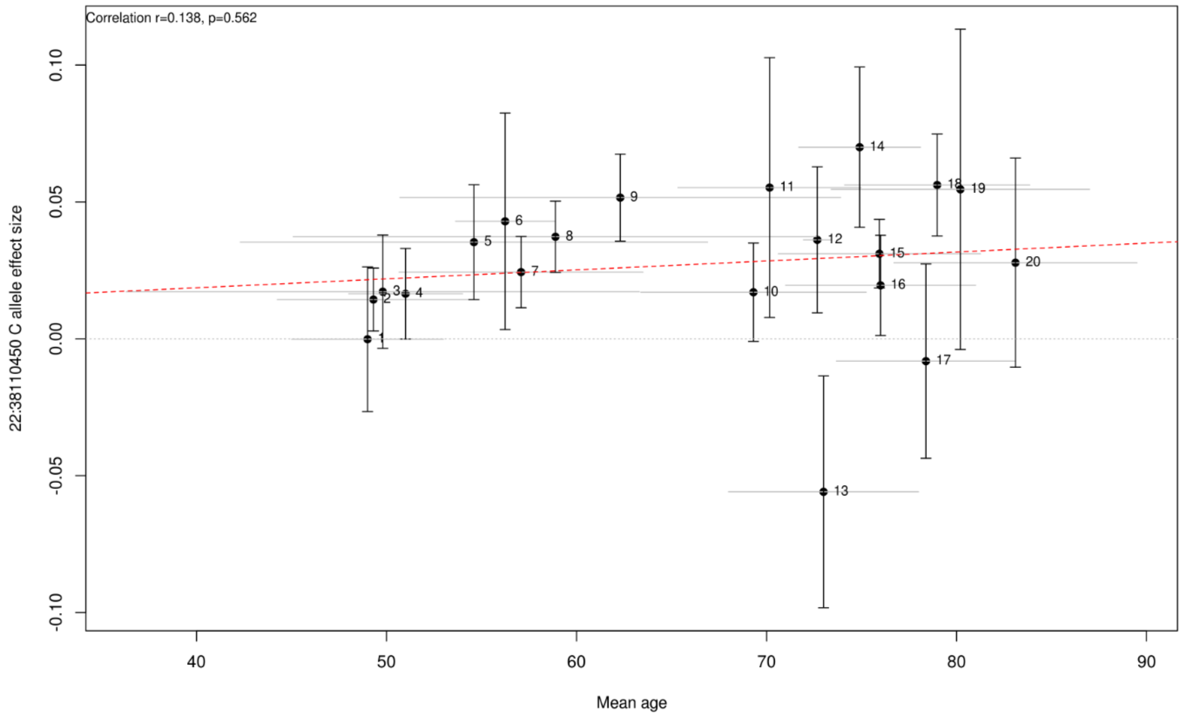

Supplementary Figure 9

Plot of effect size and mean age of cohorts with homogeneous phenotyping method for lead variant at 22q13.1 locus. Each number point denotes a cohort (1- CARDIA-AA, 2-SYS, 3-SHIP-trend, 4-CARDIA-EA, 5-SHIP, 6-VETSA, 7-RSIII, 8-FHS, 9-LIFE-Adult, 10-RSII, 11-OATS, 12-LBC1936, 13-ARIC-V5-AA, 14-PROSPER, 15-AGES, 16-ARIC-V5-EA, 17-MAS, 18-RSI, 19- RUSH-ROSMAP Batch 2, 20-RUSH-ROSMAP Batch 1). The plot includes SD of age range for each cohort.

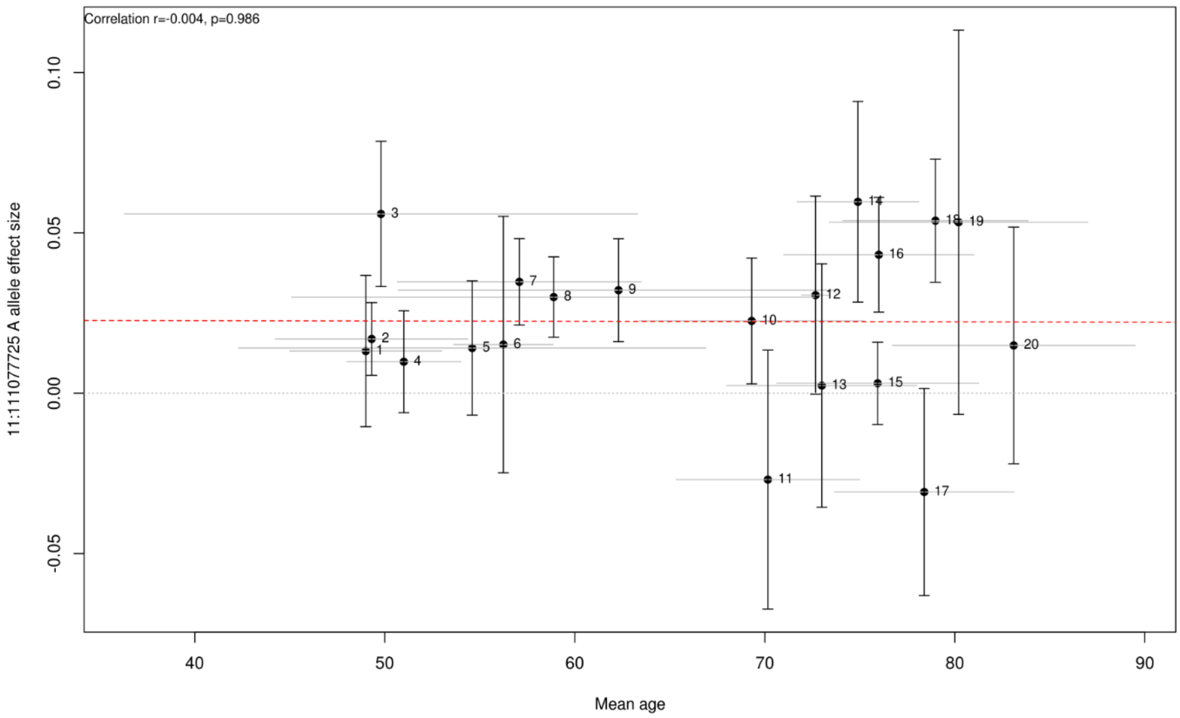

Supplementary Figure 10

Plot of effect size and mean age of cohorts with homogeneous phenotyping method for lead variant at 11q23.1 locus. Each number point denotes a cohort (1- CARDIA-AA, 2-SYS, 3-SHIP-trend, 4-CARDIA-EA, 5-SHIP, 6-VETSA, 7-RSIII, 8-FHS, 9-LIFE-Adult, 10-RSII, 11-OATS, 12-LBC1936, 13-ARIC-V5-AA, 14-PROSPER, 15-AGES, 16-ARIC-V5-EA, 17-MAS, 18-RSI, 19- RUSH-ROSMAP Batch 2, 20-RUSH-ROSMAP Batch 1). The plot includes SD of age range for each cohort.

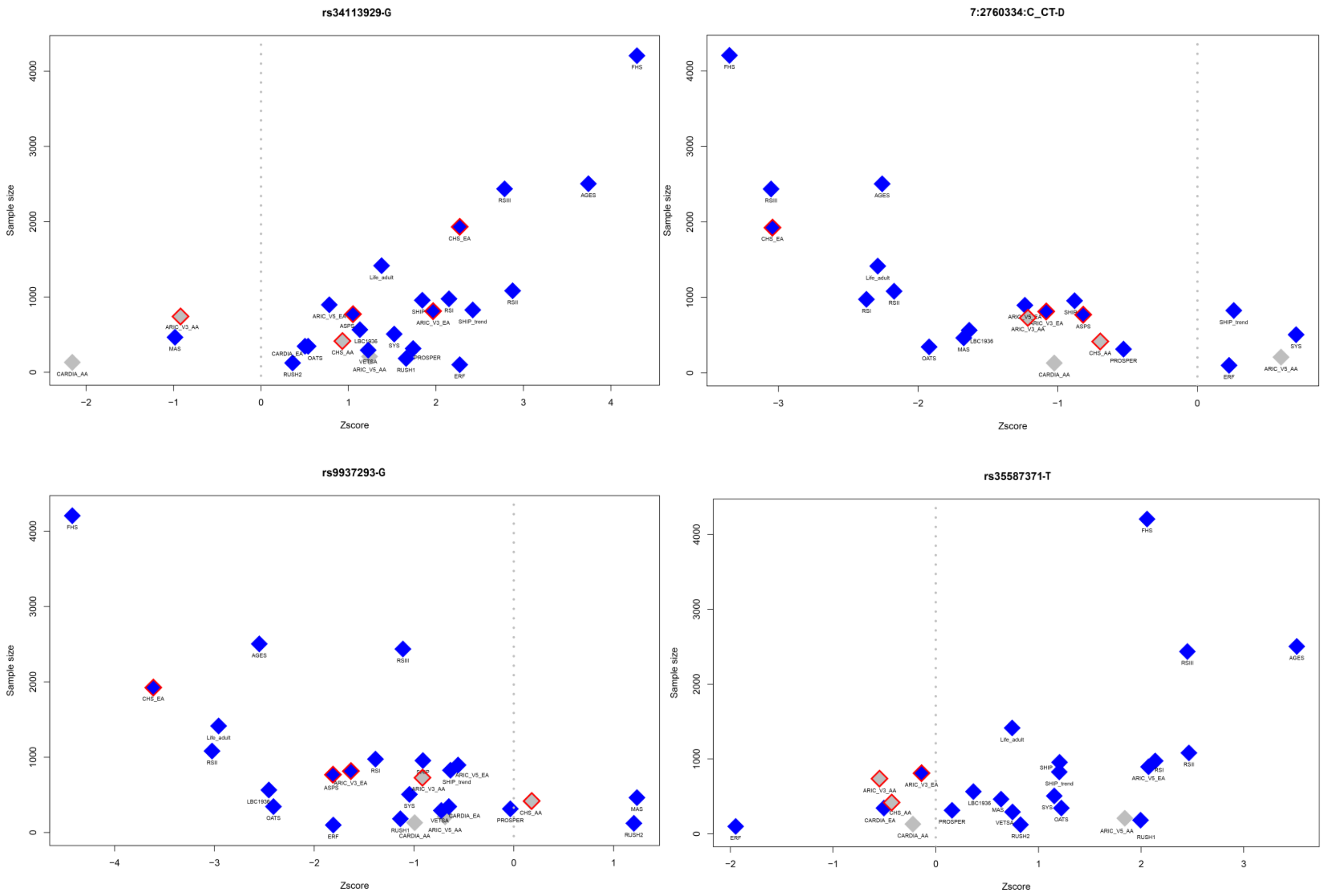

Supplementary Figure 11 (1 of 2)

Plot of Z-scores by cohort sample size for 7 lead variants. Blue diamonds denote cohorts with participants of European ancestry, whereas the gray diamonds represent cohorts with participants of African-American ancestry. Red rectangular around the diamond denotes cohorts with the visual method of phenotype assessment.

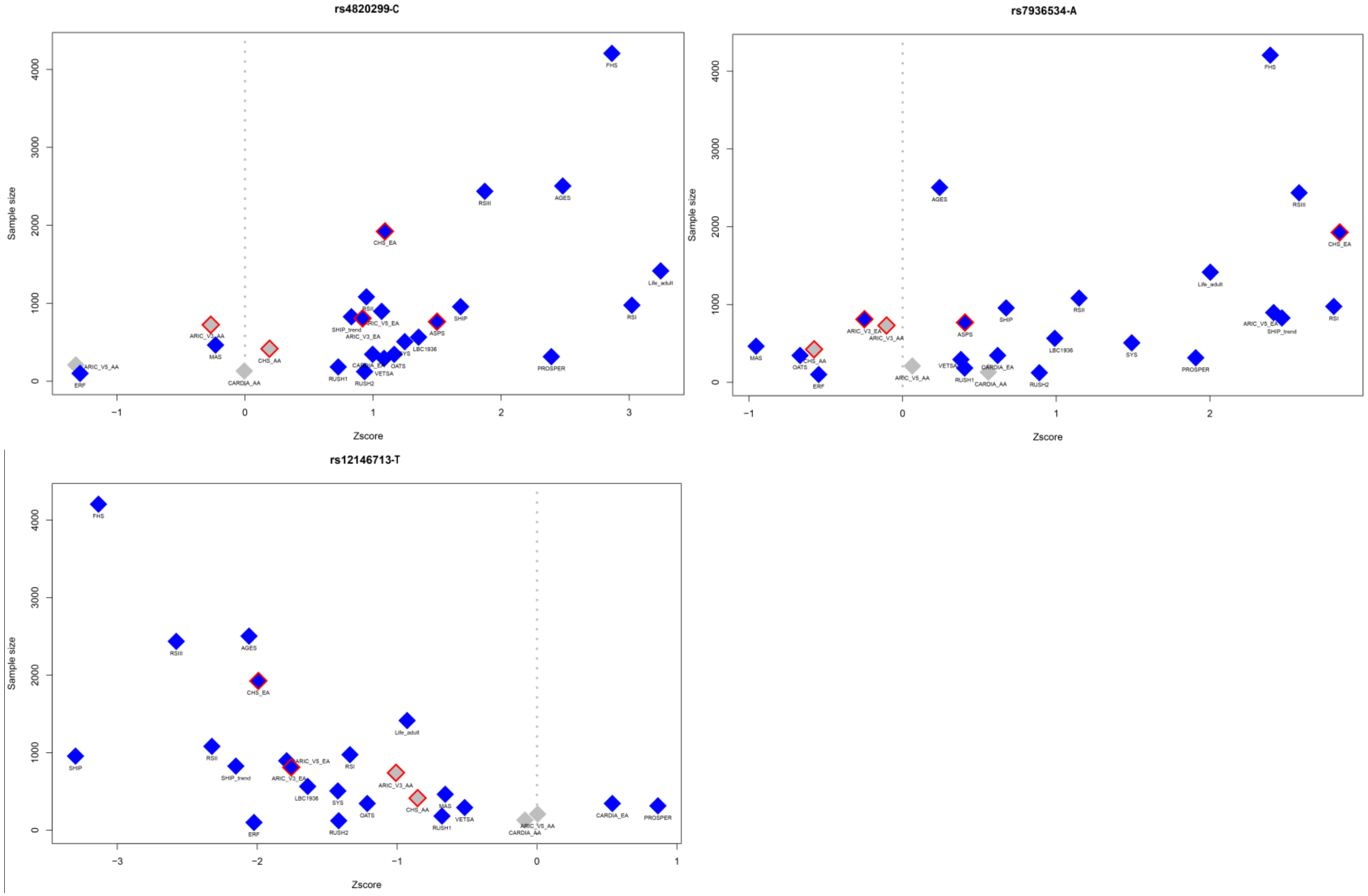

Supplementary Figure 11 (2 of 2)

Plot of Z-scores by cohort sample size for 7 lead variants. Blue diamonds denote cohorts with participants of European ancestry, whereas the gray diamonds represent cohorts with participants of African-American ancestry. Red rectangular around the diamond denotes cohorts with the visual method of phenotype assessment.

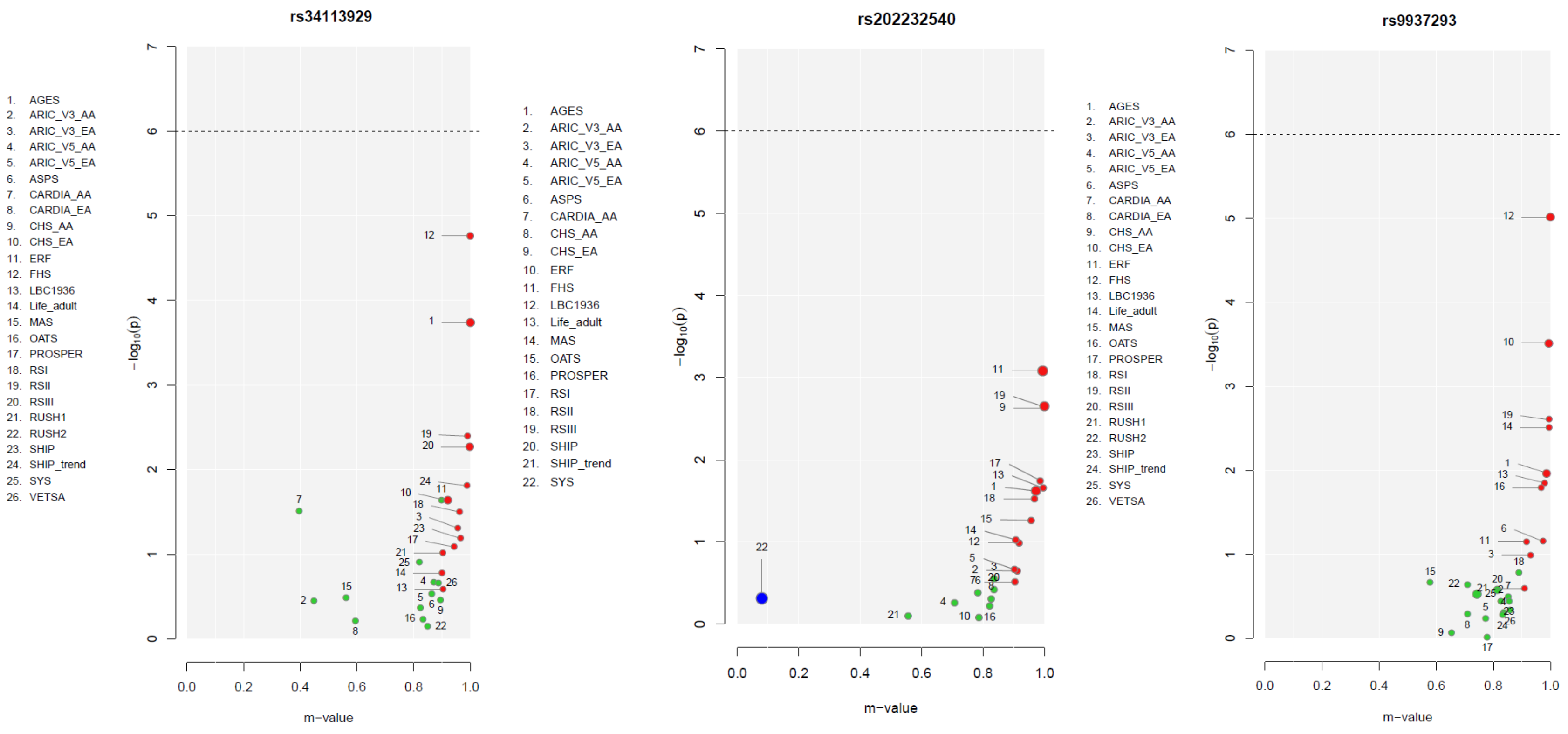

Supplementary Figure 12 (1 of 3)

PM plots for 7 lead variants. The x-axis displays the m-values (the posterior probability that the effect exists) and the y-axis represents –log10 (*p*-values) in each study. The red dots denote that the study has an effect ($m \geq 0.9$), blue dots that study does not have an effect ($m \leq 0.1$), and green dots represent studies whose effect is uncertain ($0.1 < m < 0.9$). The studies with the visual method of phenotyping include ARIC_V3_AA, ARIC_V3_EA, ASPS, CHS_EA and CHS_AA, while in other cohorts volumetric methods of phenotyping were used. The results suggest that despite heterogeneity association signals also come from cohorts that are not phenotyped in the same way, suggesting the robustness of our findings.

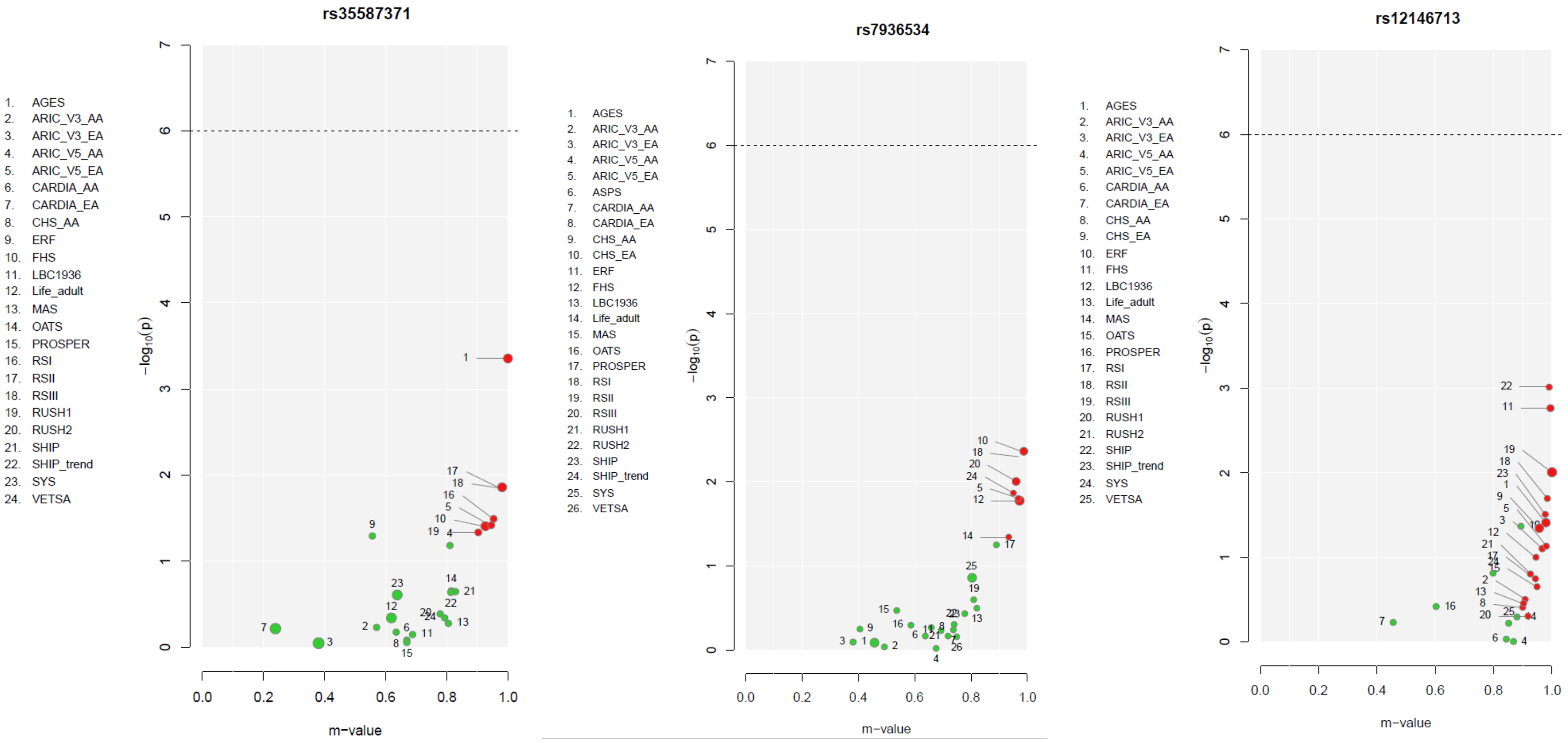

Supplementary Figure 12 (2 of 3)

PM plots for 7 lead variants. The x-axis displays the m-values (the posterior probability that the effect exists) and the y-axis represents –log10 (*p*-values) in each study. The red dots denote that the study has an effect ($m \geq 0.9$), blue dots that study does not have an effect ($m \leq 0.1$), and green dots represent studies whose effect is uncertain ($0.1 < m < 0.9$). The studies with the visual method of phenotyping include ARIC_V3_AA, ARIC_V3_EA, ASPS, CHS_EA and CHS_AA, while in other cohorts volumetric methods of phenotyping were used. The results suggest that despite heterogeneity association signals also come from cohorts that are not phenotyped in the same way, suggesting the robustness of our findings.

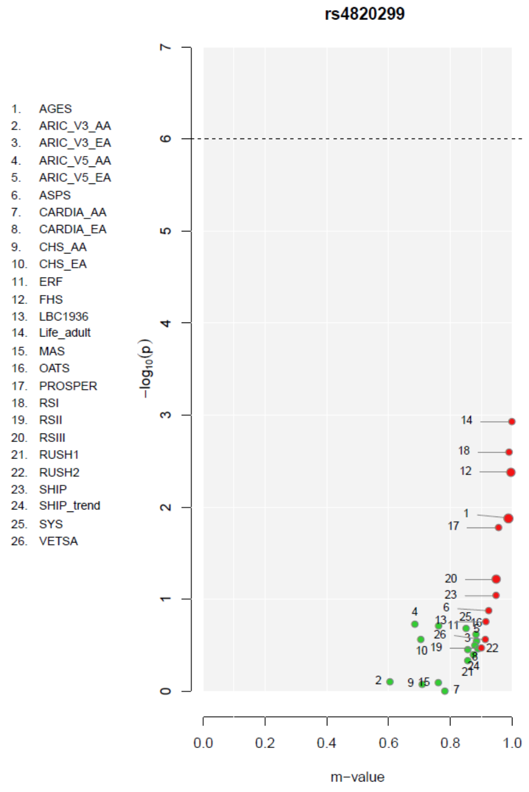

Supplementary Figure 12 (3 of 3)

PM plots for 7 lead variants. The x-axis displays the m-values (the posterior probability that the effect exists) and the y-axis represents –log10 (*p*-values) in each study. The red dots denote that the study has an effect ($m \geq 0.9$), blue dots that study does not have an effect ($m \leq 0.1$), and green dots represent studies whose effect is uncertain ($0.1 < m < 0.9$). The studies with the visual method of phenotyping include ARIC_V3_AA, ARIC_V3_EA, ASPS, CHS_EA and CHS_AA, while in other cohorts volumetric methods of phenotyping were used. The results suggest that despite heterogeneity association signals also come from cohorts that are not phenotyped in the same way, suggesting the robustness of our findings.

A.

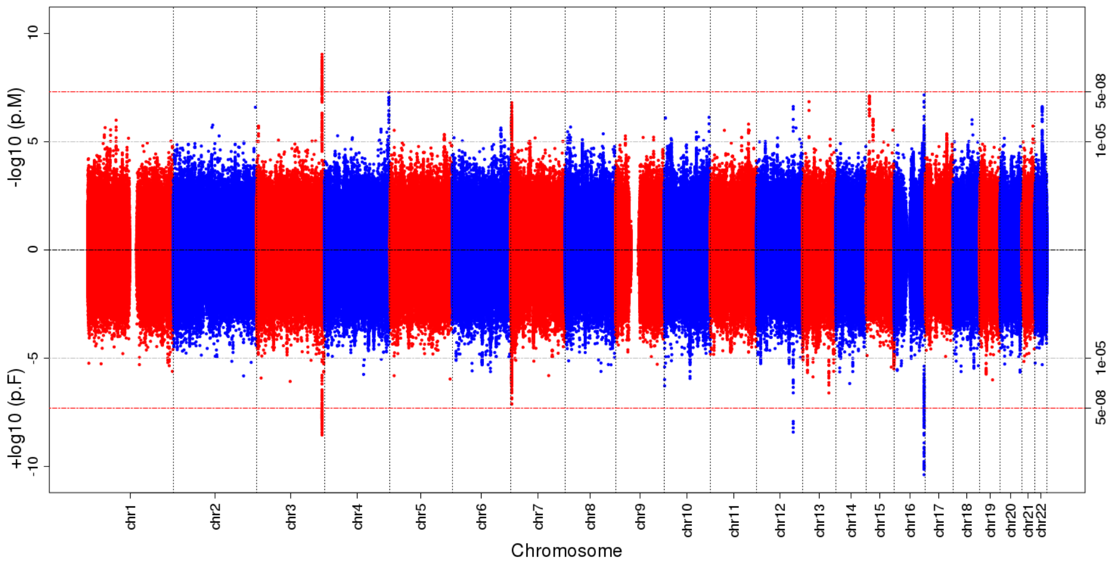

B.

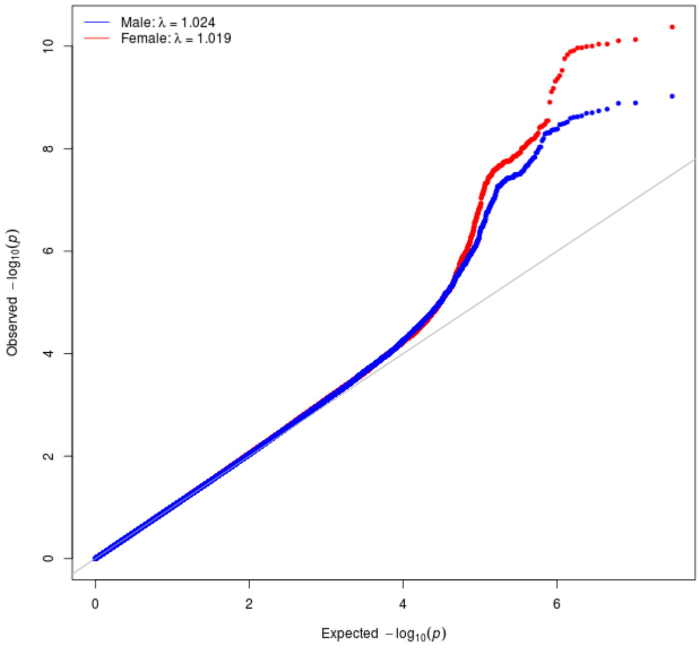

Supplementary Figure 13

A. Miami plot for sex-stratified GWA meta-analysis. Each dot represents a variant. The plot shows –log10 p-values for all variants stratified by sex (male: upper; female: lower). Red dot dash lines represent the genome-wide significance threshold (p-value < $5{\times}10^{-8}$), whereas grey dot dash lines denote suggestive threshold (p-value < $1{\times}10^{-5}$).

B. Quantile-quantile plot for sex-stratified GWA meta-analysis.

A.

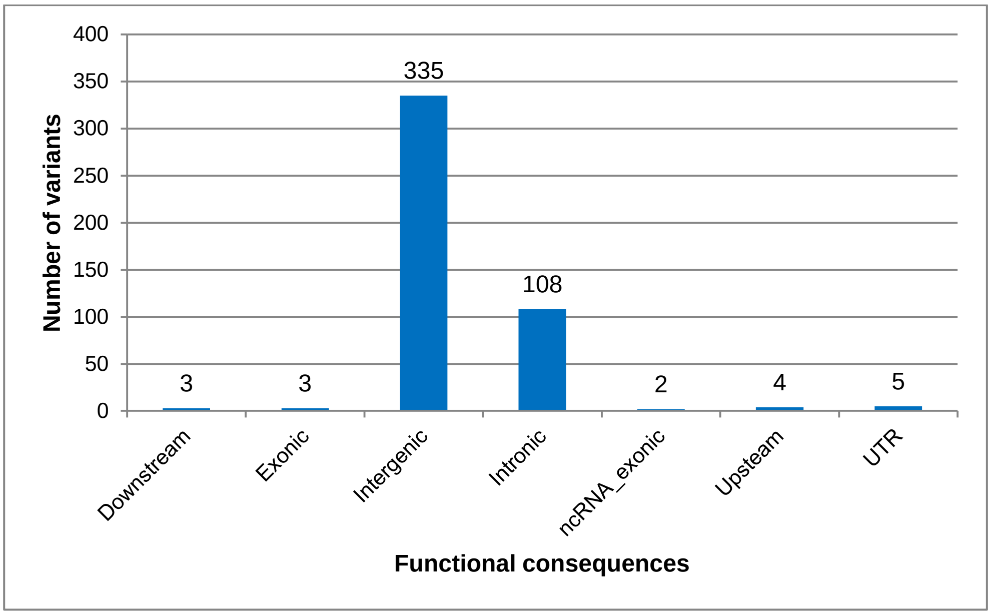

B.

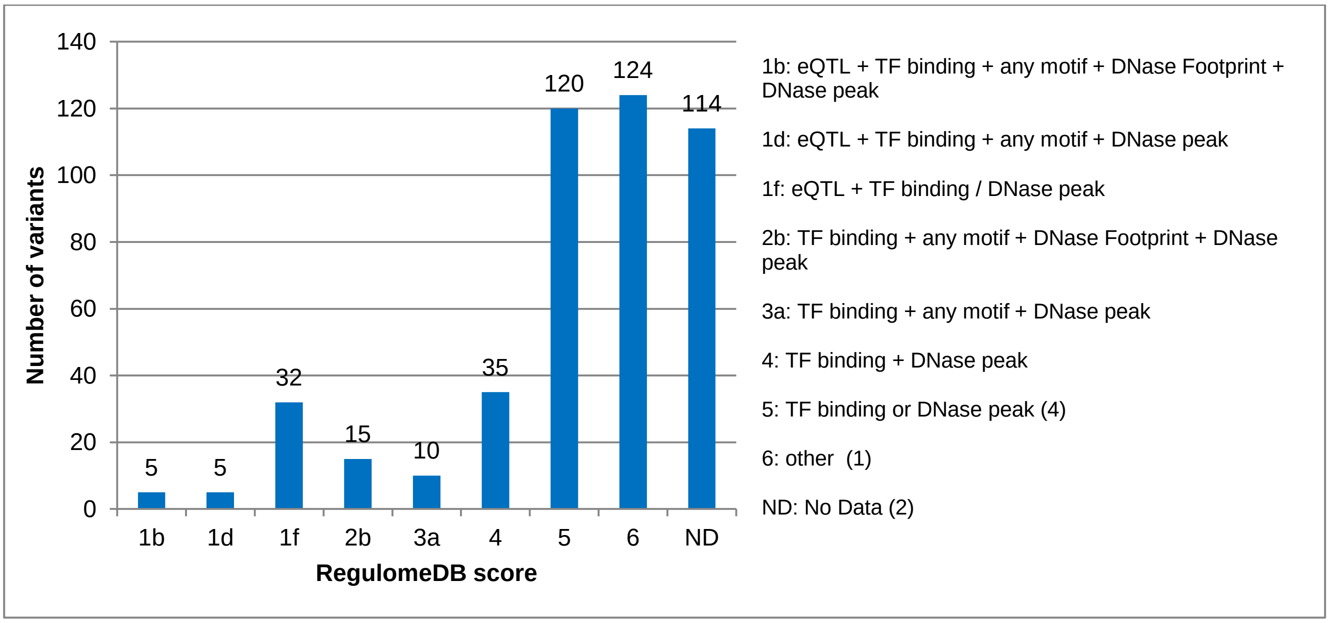

Supplementary Figure 14

Functional annotation of genome-wide significant variants from combined GWA meta-analysis (stage 3).

A. Functional consequences of all genome-wide significant variants

B. RegulomeDB score for all genome-wide significant variants. The number in parenthesis in the legend refers to the lead variants.

A. B.

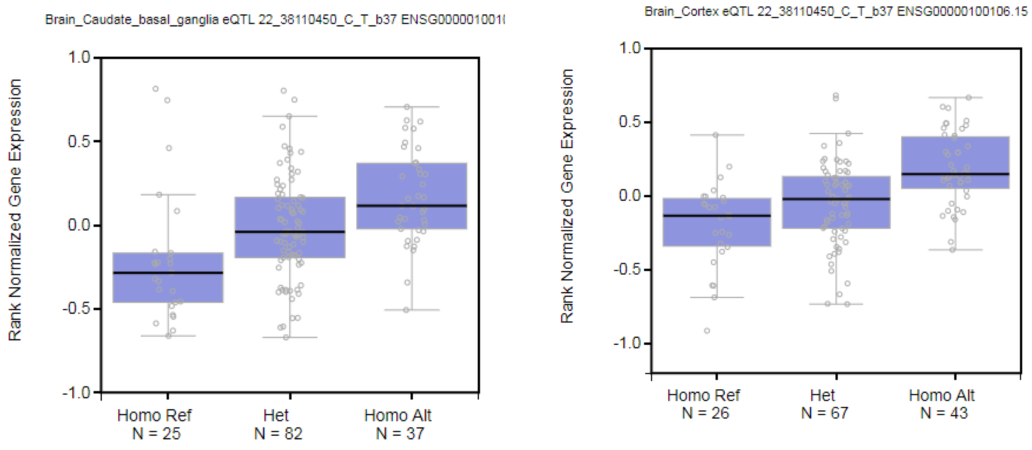

Supplementary Figure 15

The lead SNP at 22q13.1 (rs4820299) and its association with differential expression of *TRIOBP* gene in brain tissue including basal ganglia (A), and brain cortex (B).

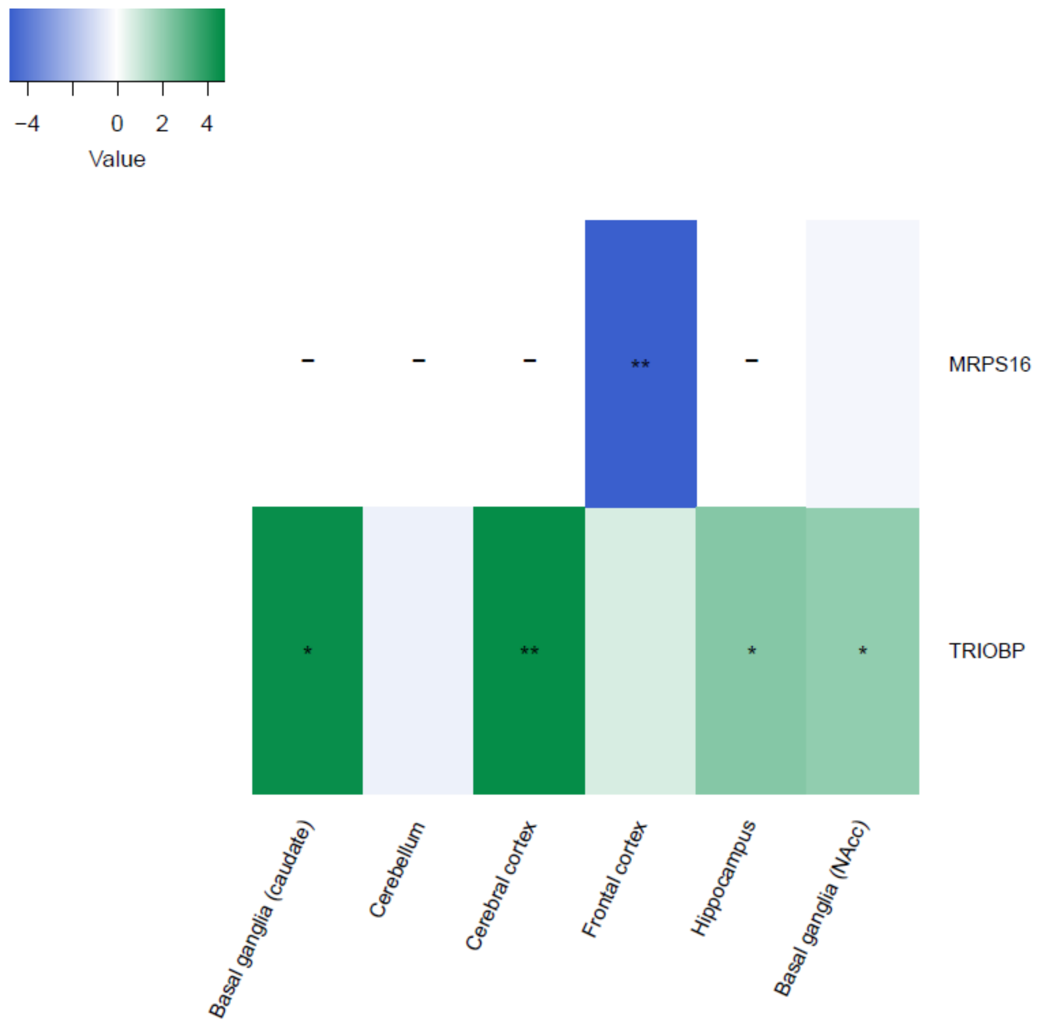

Supplementary Figure 16

Association between lateral ventricular volume and brain-specific gene-expression levels from GTEx project using MetaXcan method. Only genes for which altered expression in brain was significantly associated with lateral ventricular volume at Bonferroni corrected significance threshold are shown. Green color stands for positive correlation, while blue color stands for inverse correlation between the lateral ventricular volume and gene expression levels. Stars on the plot denote level of significance. One star stands for nominally significant association, two stars denote Bonferroni corrected *p*-value, whereas dash denotes no data available.

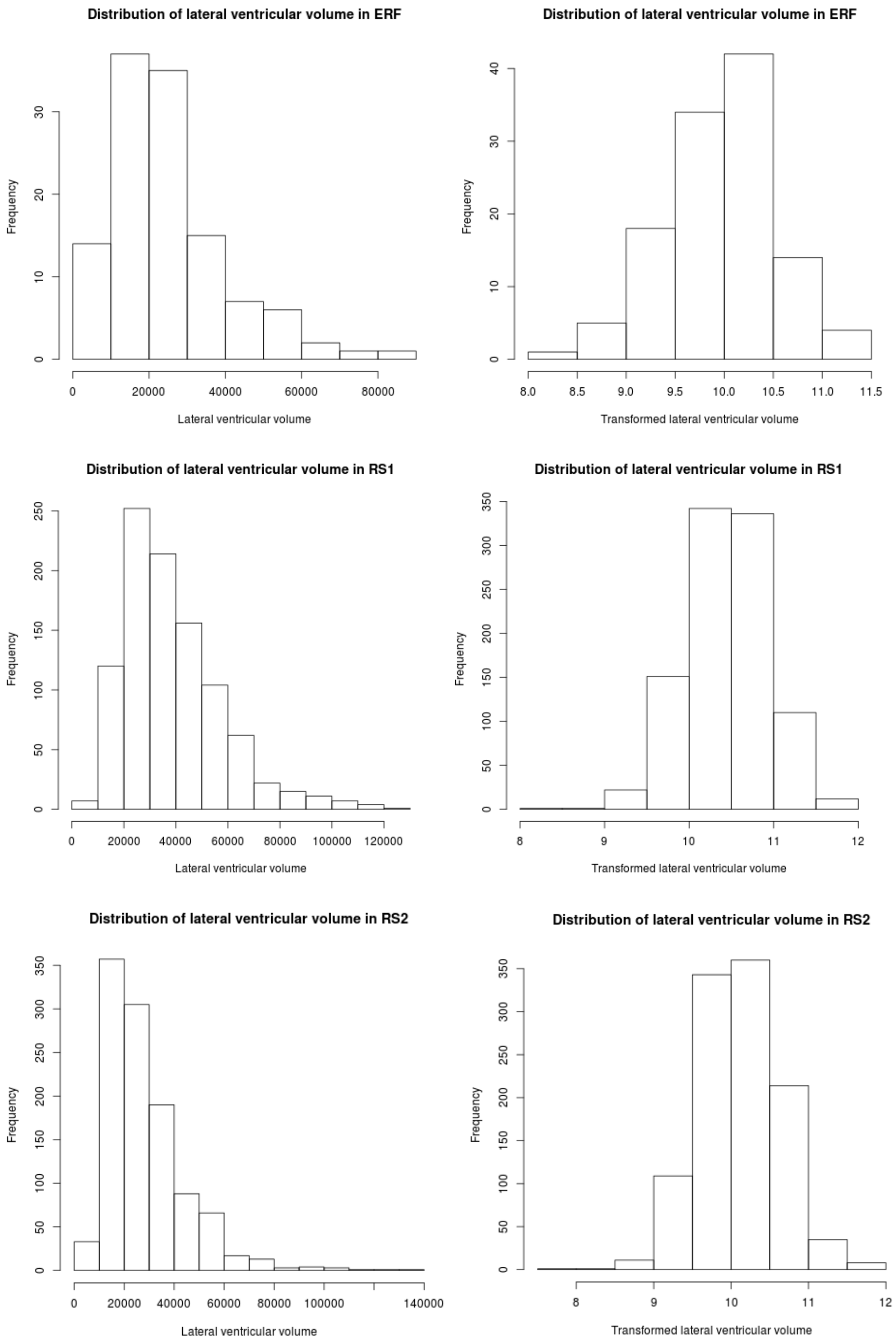

Supplementary Figure 17 (1 of 9)

Distribution plot of untransformed and transformed lateral ventricular volume in different cohorts.

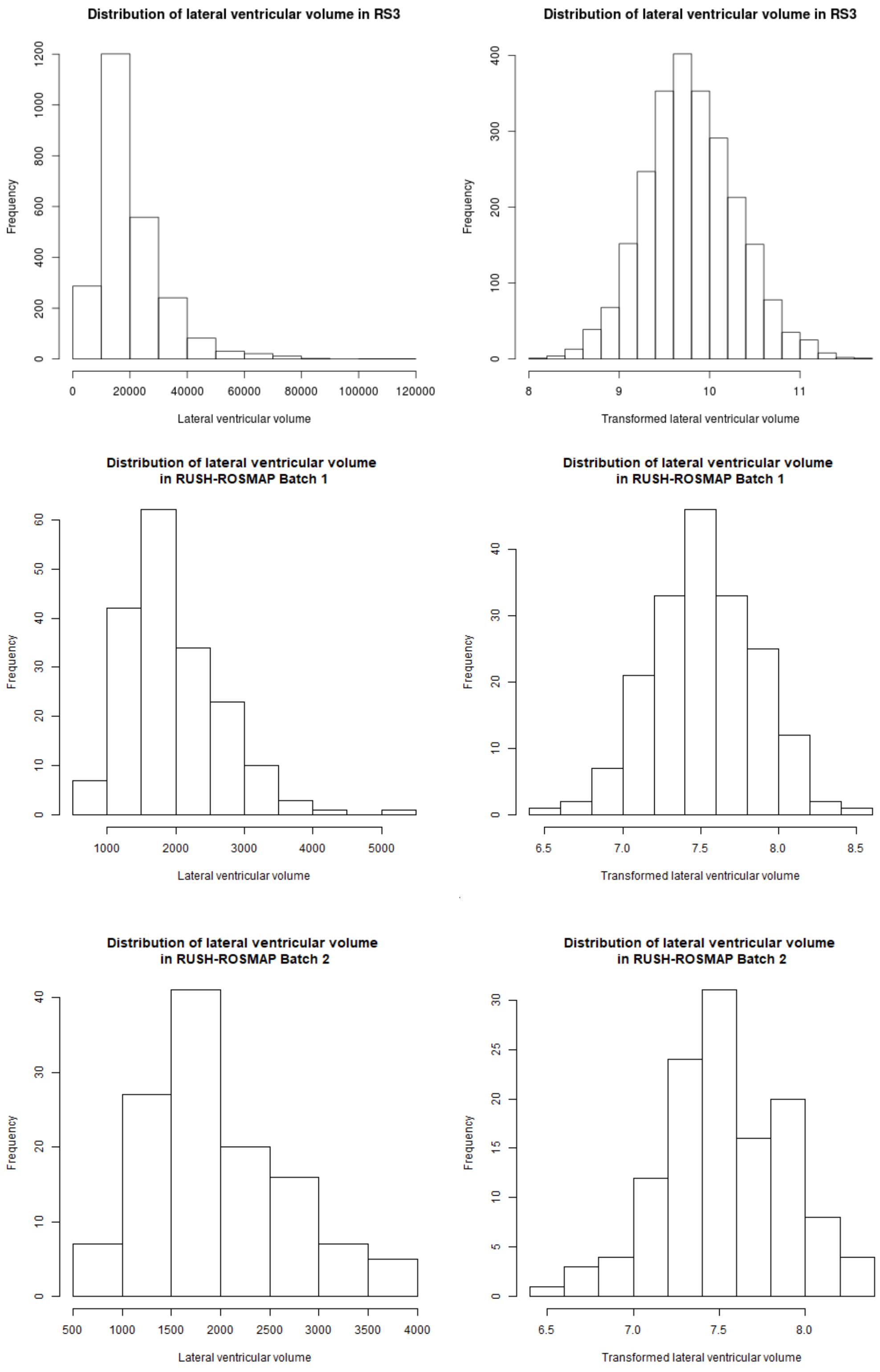

Supplementary Figure 17 (2 of 9)

Distribution plot of untransformed and transformed lateral ventricular volume in different cohorts.

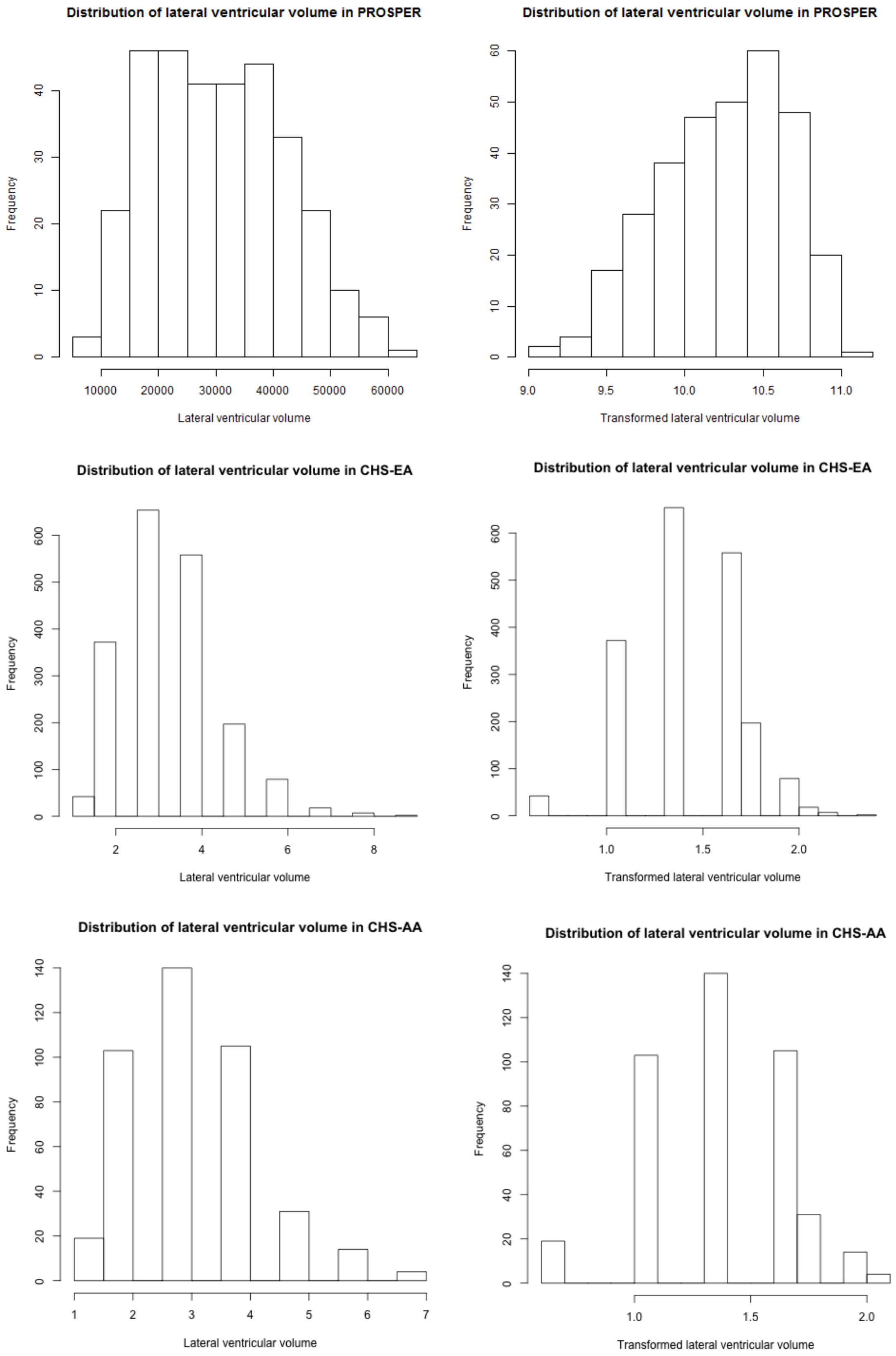

Supplementary Figure 17 (3 of 9)

Distribution plot of untransformed and transformed lateral ventricular volume in different cohorts.

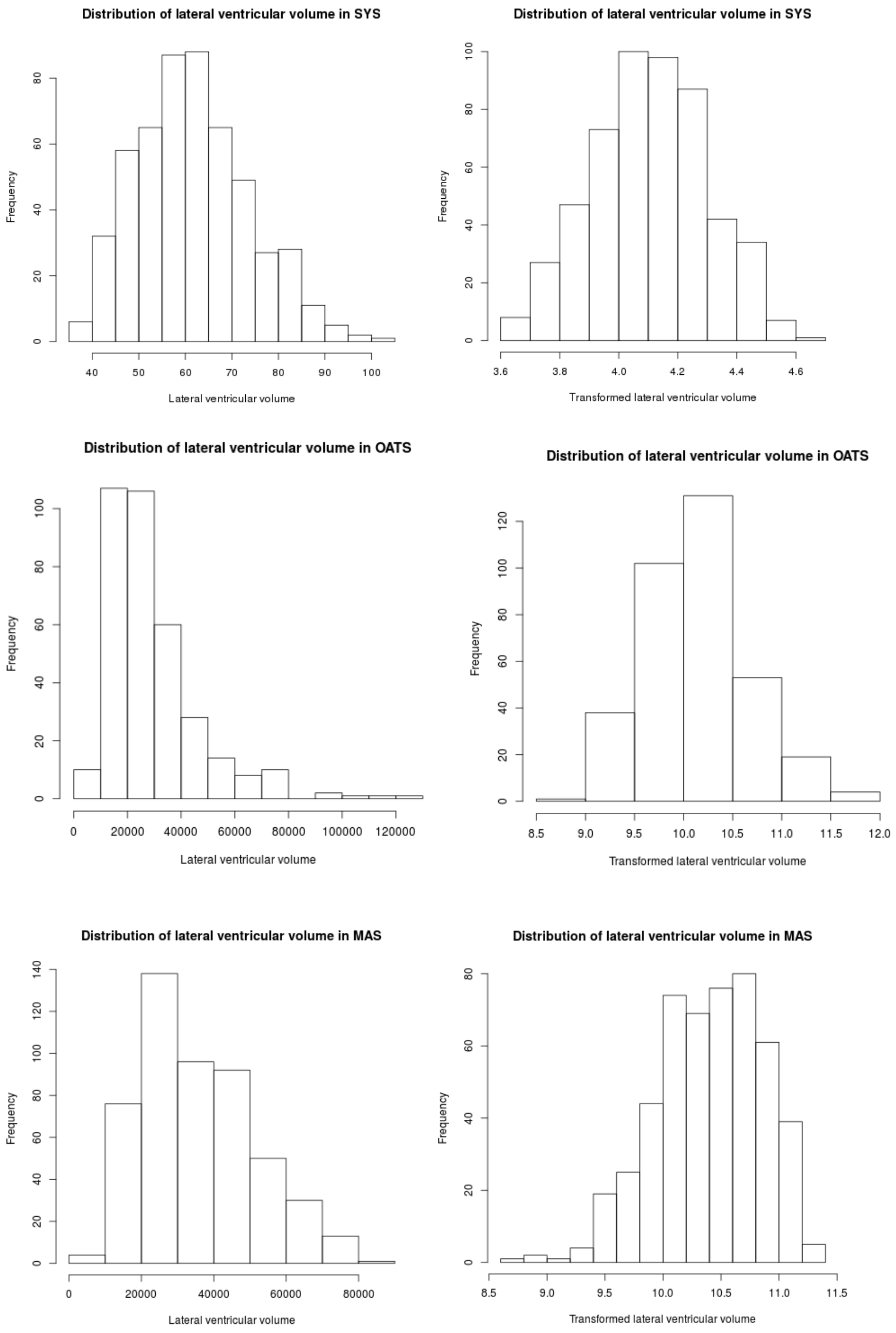

Supplementary Figure 17 (4 of 9)

Distribution plot of untransformed and transformed lateral ventricular volume in different cohorts.

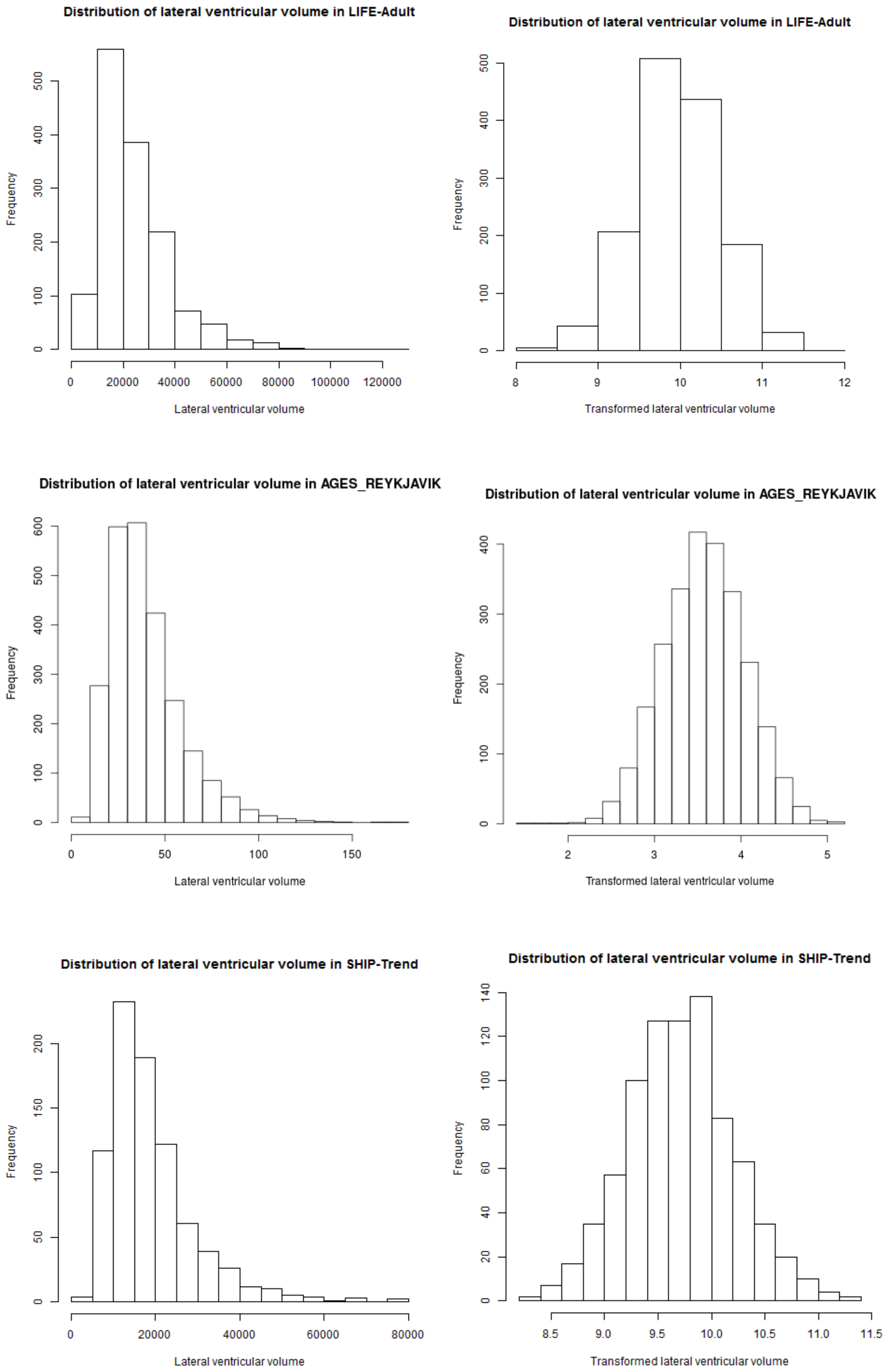

Supplementary Figure 17 (5 of 9)

Distribution plot of untransformed and transformed lateral ventricular volume in different cohorts.

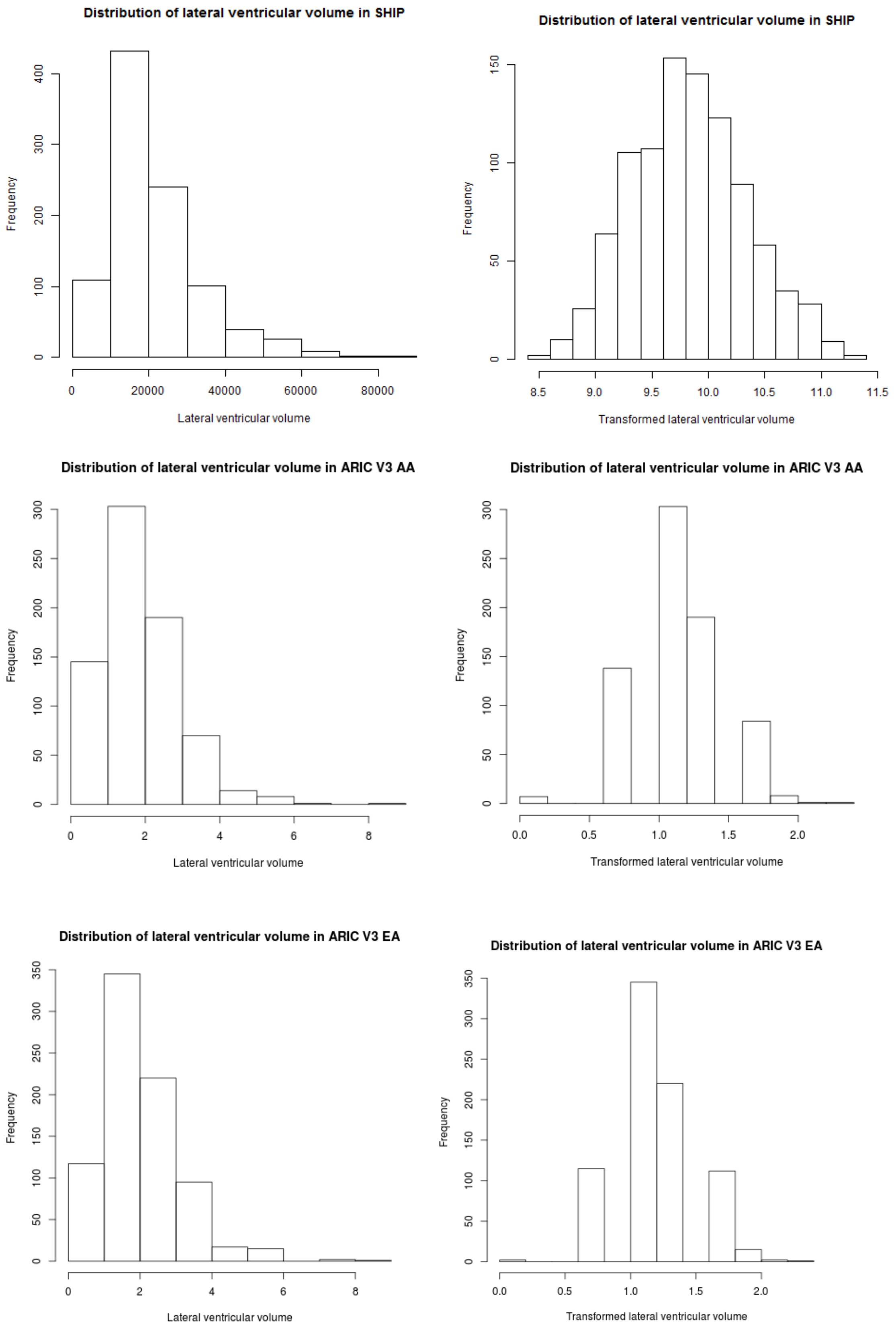

Supplementary Figure 17 (6 of 9)

Distribution plot of untransformed and transformed lateral ventricular volume in different cohorts.

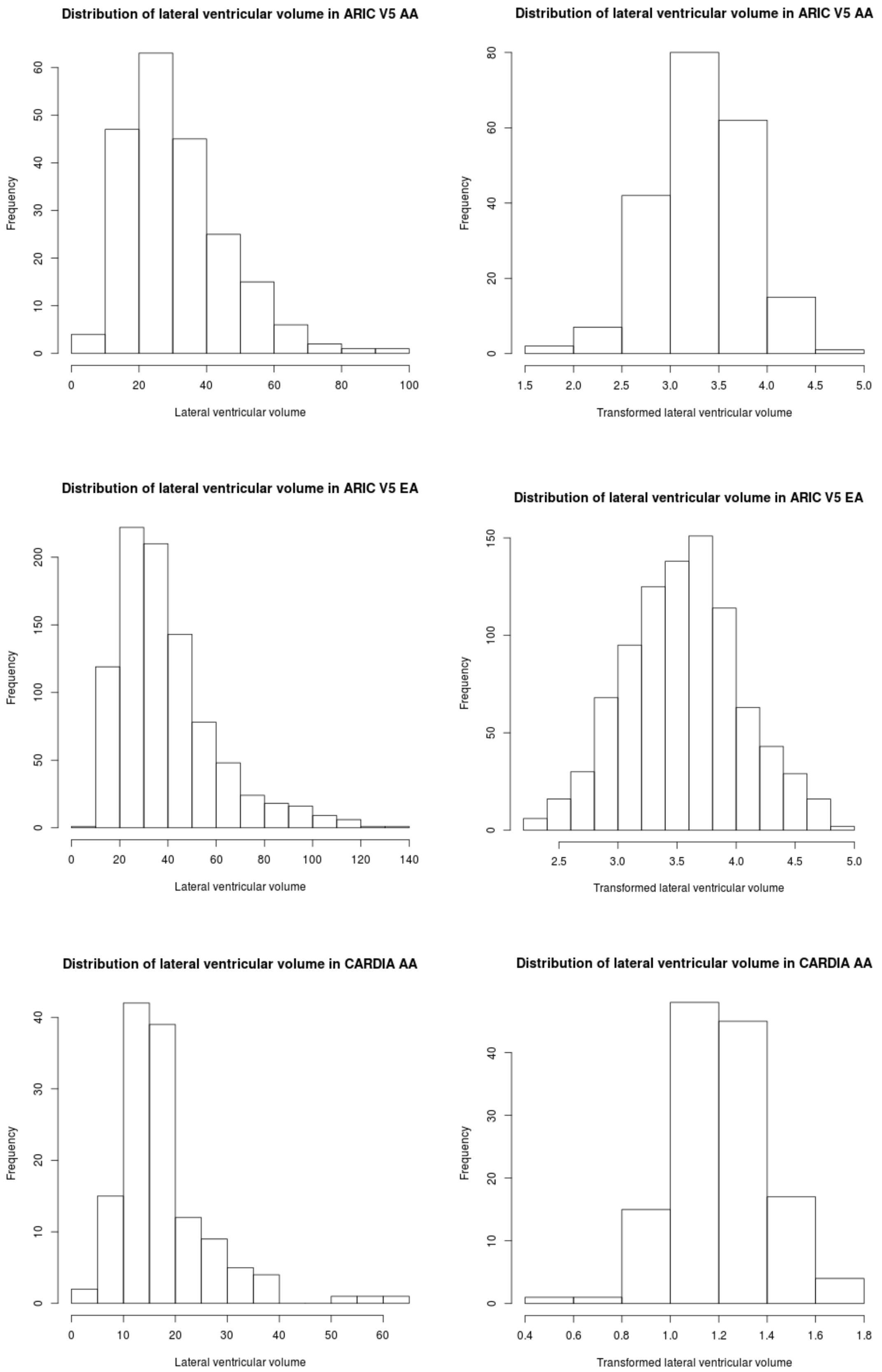

Supplementary Figure 17 (7 of 9)

Distribution plot of untransformed and transformed lateral ventricular volume in different cohorts.

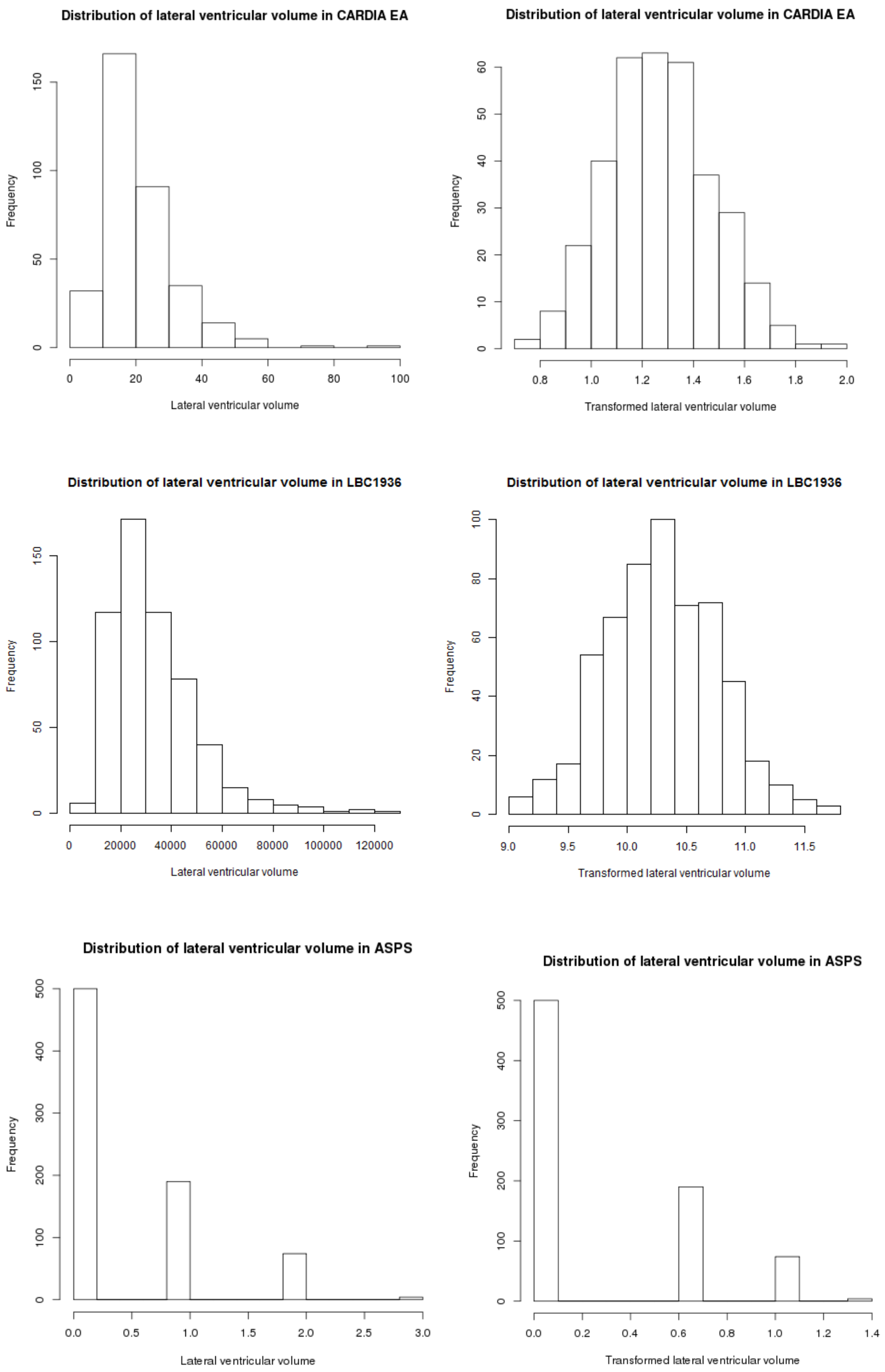

Supplementary Figure 17 (8 of 9)

Distribution plot of untransformed and transformed lateral ventricular volume in different cohorts.

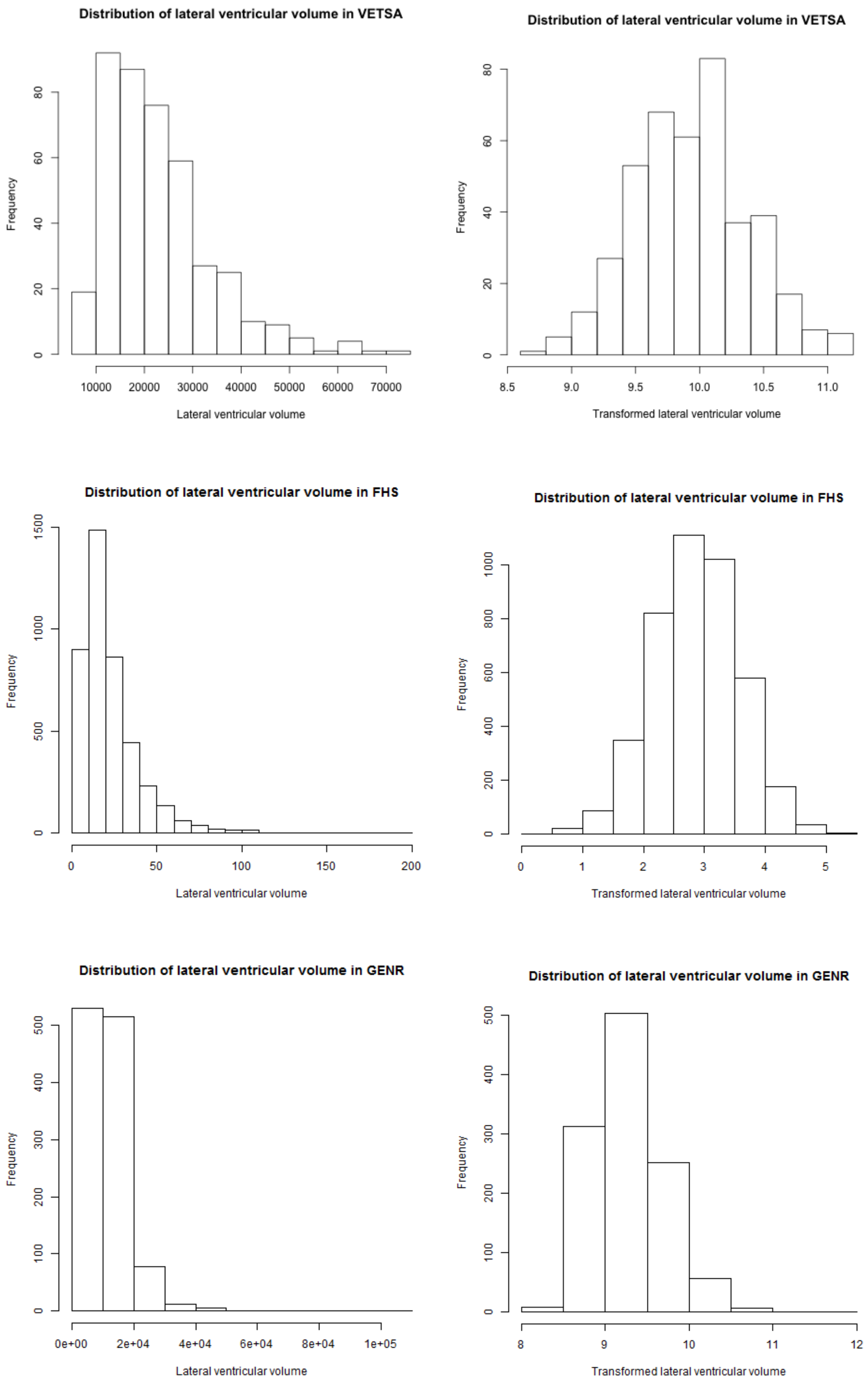

Supplementary Figure 17 (9 of 9)

Distribution plot of untransformed and transformed lateral ventricular volume in different cohorts.

Supplementary Tables

Supplementary Table 1. Genomic inflation factor for the individual studies.

Study name	Ancestry	Lambda	SE
AGES-Reykjavik	EA	1.084	0.023
ASPS	EA	0.992	0.029
CHS-EA	EA	1	0.013
CHS-AA	AA	1.002	0.035
ERF	EA	1.05	0.103
LBC1936	EA	0.998	0.042
PROSPER	EA	1.02	0.044
RSI	EA	1.011	0.033
RSII	EA	1.011	0.033
RSIII	EA	1.018	0.026
RUSH-ROSMAP Batch 1	EA	1.003	0.048
RUSH-ROSMAP Batch 2	EA	1	0.073
ARIC-V3-EA	EA	1.008	0.024
ARIC-V3-AA	AA	0.997	0.035
ARIC-V5-EA	EA	0.997	0.028
ARIC-V5-AA	AA	0.98	0.072
CARDIA-EA	EA	1.019	0.021
CARDIA-AA	AA	0.963	0.041
FHS	EA	1.006	0.025
LIFE-Adult	EA	1.014	0.034
MAS	EA	1.01	0.051
OATS	EA	1.005	0.06
SHIP	EA	1.017	0.038
SHIP-Trend	EA	1.008	0.039
SYS	EA	1.001	0.016
VETSA	EA	1.005	0.056

Supplementary Table 2. Partitioned heritability analysis performed on lateral ventricular volume dataset. Prop.SNPs refers to the proportion of SNPs that were a part of corresponding functional annotation category, whereas the prop.h2 refers to proportion of heritability attributable to corresponding functional categories.

Category	Prop. SNPs	Prop. h2	Prop._h2 std_error	Enrichment	Enrichment std_error	Enrichment p
Coding 500	0.065	0.165	0.087	2.558	1.340	2.39E-01
Conserved 500	0.333	0.693	0.131	2.083	0.393	7.91E-03
CTCF 500	0.071	0.245	0.151	3.454	2.128	2.46E-01
DGF 500*	0.542	1.255	0.199	2.318	0.367	4.21E-04
DHS 500	0.499	0.864	0.194	1.731	0.388	6.51E-02
Enhancer Andersson 500	0.019	-0.033	0.081	-1.711	4.253	5.15E-01
Enhancer Hoffman 500	0.154	0.327	0.154	2.126	1.001	2.72E-01
Fetal DHS 500	0.285	0.888	0.200	3.117	0.702	3.99E-03
H3K27ac 500	0.423	0.847	0.101	2.003	0.238	1.72E-04
H3K27ac 500	0.336	0.794	0.110	2.363	0.327	8.04E-05
H3K4me1 500	0.609	0.984	0.106	1.616	0.174	1.47E-03
H3K4me3 500	0.255	0.771	0.151	3.019	0.591	1.19E-03
H3K9ac 500	0.231	0.727	0.140	3.155	0.609	1.06E-03
Intron 500	0.397	0.552	0.062	1.391	0.156	9.86E-03
Promoter Flanking 500	0.033	0.085	0.098	2.549	2.928	5.96E-01
Promoter 500	0.039	0.071	0.060	1.834	1.545	5.88E-01
Repressed 500	0.719	0.475	0.089	0.661	0.124	7.33E-03
Super Enhancer 500	0.172	0.539	0.075	3.141	0.437	7.92E-06
TFBS 500	0.343	0.628	0.199	1.829	0.579	1.65E-01
Transcribed 500	0.763	0.907	0.117	1.189	0.154	2.12E-01
TSS 500	0.035	0.193	0.098	5.528	2.806	1.12E-01
3-prime UTR 500	0.027	0.078	0.057	2.898	2.118	3.71E-01
5-prime UTR 500	0.028	0.066	0.067	2.357	2.404	5.72E-01
Weak Enhancer 500	0.089	0.172	0.128	1.937	1.444	5.20E-01

Abbreviations: Prop - proportion; SNPs - single nucleotide polymorphism; h2 - heritability;
* when removing the additional 500 bp window, proportion of SNPs decreased from 54% to 13.8%, indicating that majority of SNPs are not at DGF;

Supplementary Table 3. The results of gene-based analysis using VEGAS2. The genes listed in the table surpassed gene-based genome-wide threshold (*p*-value = 2.08×10^{-6} (0.05/24,050)).

Chr	Gene	Locus	nSNPs	Start*	Stop*	P	TopSNP	TopSNP-P
10	*AP3M1*	10q22.2	108	75870014	75920826	1×10^{-6}	rs146128831	2.14×10^{-7}
10	*SKIDA1*	10p12.31	35	21792408	21824611	1×10^{-6}	rs10828247	3.98×10^{-8}
16	*LOC101928708*	16q24.2	171	87235720	87270035	1×10^{-6}	rs12928520	8.09×10^{-15}
17	*AMZ2P1*	17q24.1	29	62952667	62981703	1×10^{-6}	rs59490819	5.88×10^{-8}
22	*TRIOBP*	22q13.1	262	38082994	38182563	1×10^{-6}	rs4820299	1.05×10^{-10}
3	*SNAR-I*	3q28	67	1.91E+08	1.91E+08	1×10^{-6}	rs56068001	2.12×10^{-16}
7	*AMZ1*	7p22.3	318	2709155	2765070	1×10^{-6}	rs798562	1.04×10^{-12}
7	*GNA12*	7p22.3	631	2757740	2893959	1×10^{-6}	rs798562	1.04×10^{-12}
17	*GNA13*	17q24.1	57	62995406	63062920	2×10^{-6}	rs34096535	1.06×10^{-7}

Abbreviations: Chr - chromosome; nSNPs - number of SNPs; P - *p*-value; TopSNP-P - Tops SNP *p*-value;
* Start and end stop position according the hg19 assembly;

Supplementary Table 4. Phenotypic correlation between lateral ventricular volume and MRI phenotypes. Partial correlation coefficient was calculated while controlling for the total intracranial volume.

	N	Thalamus	Caudate	Putamen	Pallidum	3rd. Ventricle	4th. Ventricle	Brain Stem	Hippoca mpus	Amygd ala	CSF	Accumbens area	5th Ventricle
CHS	938	-0.337	0.207	-0.184	-0.218	0.666	0.363	-0.329	-0.477	-0.273	0.542	-0.410	0.000
RS1	959	-0.243	0.284	-0.171	-0.314	0.662	0.255	-0.305	-0.356	-0.120	0.571	-0.235	0.189
RS2	787	-0.368	0.315	-0.170	-0.348	0.688	0.312	-0.283	-0.397	-0.211	0.570	-0.246	0.116
RS3	2427	-0.358	0.176	-0.234	-0.280	0.650	0.236	-0.266	-0.355	-0.236	0.568	-0.317	0.180

Supplementary Table 5. Association of genetic risk score for neurological or psychiatric diseases with lateral ventricular volume.

Phenotype	Estimate*	SE	P
AD	0.019	0.021	3.52E-01
AD_APOE	0.011	0.009	1.80E-01
ALS	0.001	0.006	8.74E-01
bipolar	-0.010	0.006	1.04E-01
PD	0.012	0.010	1.95E-01
PSP	0.004	0.006	4.96E-01
ptau in CSF	-0.004	0.006	5.77E-01
sch	-0.003	0.007	6.10E-01
tau in CSF	-0.016	0.006	9.59E-03
wml	0.007	0.006	2.37E-01

Abbreviations: AD - Alzheimer's disease; ALS - Amyotrophic lateral sclerosis; PD - Parkinson's disease; PSP - progressive supranuclear palsy; ptau in CSF - phosphorylated tau levels in cerebrospinal fluid; tau in CSF - tau levels in cerebrospinal fluid; sch - schizophrenia; wml - white matter lesions;
* Genetic risk score has been standardized so that it has a mean of 0 and standard deviation of 1;

Supplementary Table 6. Tau SNPs used for genetic risk score analysis and their association with lateral ventricular volume.

SNP	Estimate	SE	P
rs514716	0.008	0.014	5.76E-01
rs9877502	0.045	0.010	4.76E-06
rs769449	0.006	0.014	6.42E-01

Abbreviations: P - *p*-value;

Supplementary References

1. Harris TB, *et al.* Age, Gene/Environment Susceptibility-Reykjavik Study: multidisciplinary applied phenomics. *Am J Epidemiol* **165**, 1076-1087 (2007).

2. Schmidt R, Fazekas F, Kapeller P, Schmidt H, Hartung HP. MRI white matter hyperintensities: three-year follow-up of the Austrian Stroke Prevention Study. *Neurology* **53**, 132-139 (1999).

3. Schmidt R, *et al.* Assessment of cerebrovascular risk profiles in healthy persons: definition of research goals and the Austrian Stroke Prevention Study (ASPS). *Neuroepidemiology* **13**, 308-313 (1994).

4. Fried LP, *et al.* The Cardiovascular Health Study: design and rationale. *Ann Epidemiol* **1**, 263-276 (1991).

5. Deary IJ, *et al.* The Lothian Birth Cohort 1936: a study to examine influences on cognitive ageing from age 11 to age 70 and beyond. *BMC Geriatr* **7**, 28 (2007).

6. Wardlaw JM, *et al.* Brain aging, cognition in youth and old age and vascular disease in the Lothian Birth Cohort 1936: rationale, design and methodology of the imaging protocol. *Int J Stroke* **6**, 547-559 (2011).

7. Shepherd J, *et al.* The design of a prospective study of Pravastatin in the Elderly at Risk (PROSPER). PROSPER Study Group. PROspective Study of Pravastatin in the Elderly at Risk. *Am J Cardiol* **84**, 1192-1197 (1999).

8. Shepherd J, *et al.* Pravastatin in elderly individuals at risk of vascular disease (PROSPER): a randomised controlled trial. *Lancet* **360**, 1623-1630 (2002).

9. Trompet S, *et al.* Replication of LDL GWAs hits in PROSPER/PHASE as validation for future (pharmaco)genetic analyses. *BMC Med Genet* **12**, 131 (2011).

10. Bennett DA, Schneider JA, Buchman AS, Barnes LL, Boyle PA, Wilson RS. Overview and findings from the rush Memory and Aging Project. *Curr Alzheimer Res* **9**, 646-663 (2012).

11. Bennett DA, Schneider JA, Arvanitakis Z, Wilson RS. Overview and findings from the religious orders study. *Curr Alzheimer Res* **9**, 628-645 (2012).

12. The Atherosclerosis Risk in Communities (ARIC) Study: design and objectives. The ARIC investigators. *Am J Epidemiol* **129**, 687-702 (1989).

13. Friedman GD, *et al.* Cardia - Study Design, Recruitment, and Some Characteristics of the Examined Subjects. *J Clin Epidemiol* **41**, 1105-1116 (1988).

14. Dawber TR, Kannel WB. The Framingham study. An epidemiological approach to coronary heart disease. *Circulation* **34**, 553-555 (1966).

15. Feinleib M, Kannel WB, Garrison RJ, McNamara PM, Castelli WP. The Framingham Offspring Study. Design and preliminary data. *Prev Med* **4**, 518-525 (1975).

16. Splansky GL*, et al.* The Third Generation Cohort of the National Heart, Lung, and Blood Institute's Framingham Heart Study: design, recruitment, and initial examination. *Am J Epidemiol* **165**, 1328-1335 (2007).

17. Loeffler M*, et al.* The LIFE-Adult-Study: objectives and design of a population-based cohort study with 10,000 deeply phenotyped adults in Germany. *BMC Public Health* **15**, 691 (2015).

18. Sachdev PS*, et al.* The Sydney Memory and Ageing Study (MAS): methodology and baseline medical and neuropsychiatric characteristics of an elderly epidemiological non-demented cohort of Australians aged 70-90 years. *Int Psychogeriatr* **22**, 1248-1264 (2010).

19. Sachdev PS*, et al.* A comprehensive neuropsychiatric study of elderly twins: the Older Australian Twins Study. *Twin Res Hum Genet* **12**, 573-582 (2009).

20. Pausova Z*, et al.* Cohort Profile: The Saguenay Youth Study (SYS). *Int J Epidemiol* **46**, e19 (2017).

21. John U*, et al.* Study of Health In Pomerania (SHIP): a health examination survey in an east German region: objectives and design. *Soz Praventivmed* **46**, 186-194 (2001).

22. Volzke H*, et al.* Cohort profile: the study of health in Pomerania. *Int J Epidemiol* **40**, 294-307 (2011).

23. Kremen WS*, et al.* Genes, environment, and time: the Vietnam Era Twin Study of Aging (VETSA). *Twin Res Hum Genet* **9**, 1009-1022 (2006).

24. Kremen WS, Franz CE, Lyons MJ. VETSA: the Vietnam Era Twin Study of Aging. *Twin Res Hum Genet* **16**, 399-402 (2013).

25. Kooijman MN*, et al.* The Generation R Study: design and cohort update 2017. *Eur J Epidemiol* **31**, 1243-1264 (2016).
