# Supplementary material for: Genome-wide association study of 23,500 individuals identifies 7 loci associated with brain ventricular volume
Source: Nat Commun. 2018 Sep 26;9:3945. doi: 10.1038/s41467-018-06234-w (PMC6158214; doi:10.1038/s41467-018-06234-w)
Supplement: Supplementary file 3 — Description of Additional Supplementary Files [file 41467_2018_6234_MOESM3_ESM.pdf]

Description of Additional Supplementary Files

File Name: Supplementary Data 1
Description: Demographic characteristic of study population.

File Name: Supplementary Data 2
Description: Information on image acquisition and processing.

File Name: Supplementary Data 3
Description: Information on genotyping and analysis.

File Name: Supplementary Data 4
Description: Genome-wide significant variants and their corresponding p-values across the three stages and children's cohort. The lead variant at each of genome-wide significant locus is denoted with a star.

File Name: Supplementary Data 5
Description: Association of lead SNPs and their proxies (r 2 > 0.7, p-value < 5×10- 8) with various traits and diseases according to PhenoScanner database

File Name: Supplementary Data 6
Description: Annotation of all genome-wide significant variants in the combined meta-analysis (stage 3). The lead variant for each genome-wide significant locus is denoted with a star.

File Name: Supplementary Data 7
Description: Functional enrichment analysis of lateral ventricular volume loci using GARFIELD.

File Name: Supplementary Data 8
Description: Top results of pathway-based analysis (p-value < 5×10-5).

File Name: Supplementary Data 9
Description: Genes part of the “regulation of cytoskeleton organization” pathway and diseases they have been implicated in according to the DisGeNET database. The associations supported by at least two curated databases (Score > 0.2) are shown.

File Name: Supplementary Data 10
Description: List of lead SNPs extracted from the largest published GWAS for genetics risk score analysis.
